# Supercritical CO_2_ Activation Enables an Exceptional Methanol Synthesis Activity Over the Industrial Cu/ZnO/Al_2_O_3_ Catalyst

**DOI:** 10.1002/advs.202500118

**Published:** 2025-03-07

**Authors:** Yannan Zhou, Jingyun Jiang, Yushun Wang, Ruijie Liu, Shouren Zhang, Jianfang Wang

**Affiliations:** ^1^ Henan Provincial Key Laboratory of Nanocomposites and Applications Institute of Nanostructured Functional Materials Huanghe Science and Technology College Zhengzhou Henan 450006 China; ^2^ Department of Physics The Chinese University of Hong Kong Shatin Hong Kong SAR 999077 China; ^3^ College of Materials Science and Engineering Zhengzhou University Zhengzhou Henan 450052 China; ^4^ School of Chemistry and Chemical Engineering Henan University of Science and Technology Luoyang Henan 471023 China

**Keywords:** dual‐response pathway, methanol synthesis, strong metal–support interaction, supercritical CO_2_ activation, ternary Cu/ZnO/Al_2_O_3_ catalyst

## Abstract

The ternary Cu/ZnO/Al_2_O_3_ catalyst is widely used in the industry for renewable methanol synthesis. The tenuous trade‐off between the strong metal–support interaction (SMSI)‐induced Cu–ZnO*_x_* interface and the accessible Cu surface strongly affects the activity of the final catalyst. Successes in the control of oxide migration on adsorbate‐induced SMSI catalysts have motivated this to develop a supercritical CO_2_ activation strategy to synchronously perfect the Cu^0^–O–Zn*^δ^* ^+^ interface and Cu^0^–Cu^+^ surface sites through the manipulation of the adsorbate diffusion kinetics, which involves ^*^OC_2_H_5_ and “side‐on” fixed CO_2_ species. This findings illustrate that the adsorbate on ZnO*_x_* can facilitate its secondary uniform nucleation and induce a Zn*_x_* Al_2_O*_y_* spinel phase and that CO_2_ adsorption on metallic Cu^0^ produces an activated Cu*_x_* O amorphous shell. Such a structural evolution unlocks a dual‐response pathway in methanol synthesis, thus enabling Cu/ZnO/Al_2_O_3_ with a twofold increase in catalytic activity. This atomic‐level design of active sites and understanding of supercritical CO_2_‐induced structural evolution will guide the future development of high‐performance supported metal catalysts.

## Graphical abstract

A supercritical CO_2_ activation strategy is demonstrated for the ternary industrial Cu/ZnO/Al_2_O_3_ catalyst to synchronously perfect the strong metal–support interaction‐induced Cu–ZnO*_x_* interfacial and exposed Cu surface sites by manipulating the adsorbent diffusion kinetics. A dual‐response pathway in methanol synthesis is unraveled. It endows the ternary catalyst with a remarkable two‐fold increase in the catalytic activity.

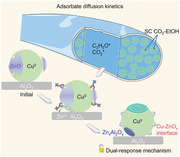

## 1 Introduction

Methanol synthesis from captured off‐gas CO_2_ and non‐fossil hydrogen through a one‐step catalytic process is the heart of the Carbon Neutral Methanol Economy.^[^ ^1^ ^]^ The leading industrial methanol catalyst, ternary Cu/ZnO/Al_2_O_3_ with Cu,Zn‐containing nanoparticles (5–10 nm in size, molar ratio Cu:Zn = 7:3) supported on the structural promoter Al_2_O_3_,^[^ ^2^ ^]^ has been producing ≈110000 tons of renewable CH_3_OH and thus recycling ≈150000 tons of CO_2_ per year.^[^ ^3^ ^]^ Although this catalytic process has been studied for centuries, the synergistic effect of Cu and ZnO and the dynamic nature of Zn species are still under high debate.^[^ ^4^ ^]^ Studies have shown that the active Cu^0^–O–Zn*^δ^* ^+^ interface created by the migration of reducible ZnO*_x_* over the metallic Cu nanoparticle under strongly reducing reaction conditions,^[^ ^5^ ^]^ the so‐called strong metal support interaction (SMSI), and the accessible Cu surface, are essential to the catalytic performance. This, therefore, highlights the significance of a highly defective or porous oxide overlayer in the SMSI effect. This configuration, where the oxide partially covers the metal nanoparticle, allows reactants to interact with most exposed metal sites.^[^ ^6^ ^]^

Nevertheless, the uncontrollable kinetics of the SMSI are insufficient to create an optimal SMSI encapsulation state.^[^ ^7^ ^]^ The ZnO*_x_* overlayer might cover all metal sites or affect only a minor portion of the interfacial sites. In addition, the dynamic nature of the ZnO*_x_* component at elevated pressures and temperatures could lead to the excessive growth and partial crystallization of ZnO*_x_*.^[^ ^8^ ^]^ This will potentially reduce the density of the interfacial active sites or cause catalyst deactivation.^[^ ^9^ ^]^ Recent progress has demonstrated that hydrocarbon species derived from CO_2_ gas, and reductive alcohols, such as methanol, can lead to adsorbate‐induced SMSI (A‐SMSI) on the catalyst under mild conditions. The resultant encapsulating overlayer is usually permeable to reactant gases.^[^ ^10^ ^]^ One important key to such an A‐SMSI phenomenon is the control of the adhesive properties of alcohols, which have been observed to correlate with the migration of oxide species.^[^ ^11^ ^]^ Highly adhesive EtOH tends to be physisorbed on the surface of the defective oxide, leading to the partial or complete poisoning of active sites and preventing the migration of oxide species. These investigations on the A‐SMSI effect and how it affects the migration ability of oxide species have intrigued us and led us to develop an activation strategy to optimize the SMSI‐induced catalyst structure through the control of the adsorbate kinetics.

Herein we demonstrate that the adsorbate diffusion kinetics on the Cu/ZnO/Al_2_O_3_ catalyst can be manipulated in a density‐fluctuating supercritical (SC) CO_2_–EtOH medium at a low temperature of 40 °C. Multiple in situ spectroscopy and microscopy show that controllable adsorbate diffusion results in the synchronous perfecting of SMSI‐induced Cu^0^–O–Zn*^δ^* ^+^ interface and exposed Cu^0^–Cu^+^ surface active sites. Specifically, adsorbed ^*^OC_2_H_5_ and CO_2_ species on the support guide the secondary nucleation of small‐sized ZnO on the Cu surface and the formation of a new Zn*_x_* Al_2_O*_y_* phase, while CO_2_ adsorption on metallic Cu^0^ yields a large accessible activated Cu*_x_* O surface area. We reveal that such an activation treatment renders the Cu/ZnO/Al_2_O_3_ catalyst resistant to the Cu sintering and ZnO*_x_* recrystallization under working conditions. Moreover, it facilitates a dual‐response pathway in the methanol synthesis from CO_2_ hydrogenation, involving (i) formate and (ii) reverse water gas shift (RWGS) + CO hydrogenation mechanisms, thus improving the activity, selectivity, and stability. Under optimal conditions, the 12 MPa (4 h) catalyst exhibits the highest mass‐specific methanol formation rate of 259.09 g kg_cat_^−1^ h^−1^ at 210  °C, which is 2.1 times higher than that of the industrial catalyst (125.87 g kg_cat_^−1^ h^−1^). Our results show that controlling the kinetics of adsorbate diffusion represents a powerful support effect that enables the rational manipulation of the catalyst structure.

## 2 Results and Discussion

### 2.1 Catalytic Performance

**Figure 1:**
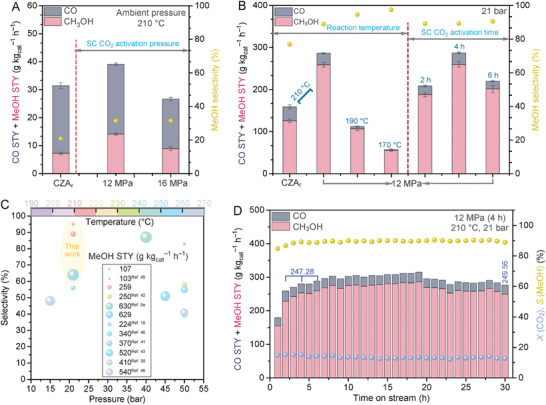
Performances of the different catalysts in CO_2_ hydrogenation to methanol. A) MeOH selectivities and STYs of the differently activated catalysts for the reactions at 210  °C and atmospheric pressure. B) MeOH selectivities and STYs for the reactions at 170–210  °C and 21 bar. In all cases, results are expressed as mean ± SEM (*n* = 3), and significance was defined as *p* < 0.05. C) Performance comparison between the 12 MPa (4 h) catalyst and other reported Cu‐based catalysts for methanol production from CO_2_ hydrogenation. D) Stability test of the 12 MPa (4 h) catalyst over 30 h. The catalyst was pre‐treated in situ with H_2_ at 350 °C for 1 h. Reaction conditions: molar ratio CO_2_:H_2_ = 1:3, 20 mg catalyst, flow rate 20 mL min^−1^. The reaction activity tests were conducted in a Harrick reactor.

The reduced industrial CuO/ZnO/Al_2_O_3_ catalyst, hereafter referred to as CZA_r_ (see Experimental Section in the Supporting Information for the details), was subjected to different activation treatments in the SC CO_2_–EtOH system and subsequently evaluated for the CO_2_ hydrogenation reaction in a Harrick reactor under pressurized (21 bar) and atmospheric pressure conditions (Figure S1, Supporting Information). The activated catalysts are denoted as *X* MPa (*Y* h), with *X* and *Y* being the activation pressure and time in the SC CO_2_–EtOH system, respectively. **Figure** **1**, Figures S2 and S3 (Supporting Information) display the steady‐state reactivity data of our catalysts at different temperatures. The catalysts were pre‐treated in situ with H_2_ before the methanol synthesis reaction. To differentiate the hydrogen reduction of the catalyst during the preparation process, the in situ H_2_ treatment is designated as H_2_ treatment^②^. CO was the only detected gaseous byproduct for our catalysts.

Significant enhancement in MeOH production was observed over the activated samples at atmospheric pressure (Figure 1A). The MeOH space time yield (STY) maximized at 14.18 g kg_cat_^−1^ h^−1^ over the 12 MPa (4 h) sample and decreased to 8.96 g kg_cat_^−1^ h^−1^ when the SC CO_2_ pressure was raised to 16 MPa. In addition, the methanol selectivity slightly increased from 20% to 32% after activation. The activities of the catalysts were further tested at 21 bar and temperatures from 170 to 250 °C (Figure 1B and Figure S2, Supporting Information). The MeOH STY increased from 55.96 to 629.28 g kg_cat_^−1^ h^−1^ as the temperature was raised, delivering a superior MeOH yield (259.09 g kg_cat_^−1^ h^−1^ at 210 °C) in comparison with CZA_r_ (125.87 g kg_cat_^−1^ h^−1^ at 210 °C) and other reported state‐of‐the‐art catalysts (Figure 1C and Table S1, Supporting Information). Notably, the most active catalyst (12 MPa (4 h)) in our study exhibited a much higher selectivity (89%) toward MeOH at 210 °C, with a high CO_2_ conversion of 14.75%. Moreover, changing the reducing gas conditions (250 °C, 2 h) had a negligible effect on the activity of the catalyst (Figure S2, Supporting Information), while the pre‐activation time of SC CO_2_ was directly related to the activity of the catalyst. The temporal evolution of the MeOH production of the 12 MPa (4 h) sample at higher temperatures (210 and 250 °C) for 30 h is displayed in Figure 1D and Figure S3 (Supporting Information), respectively, featuring a superior stability. In addition, the fresh 12 MPa (4 h) sample without H_2_ treatment^②^ also showed a higher MeOH production than the CZA_r_ catalyst, even under atmospheric pressure (Figure S4, Supporting Information).

### 2.2 Catalyst Structural Evolution

**Figure 2:**
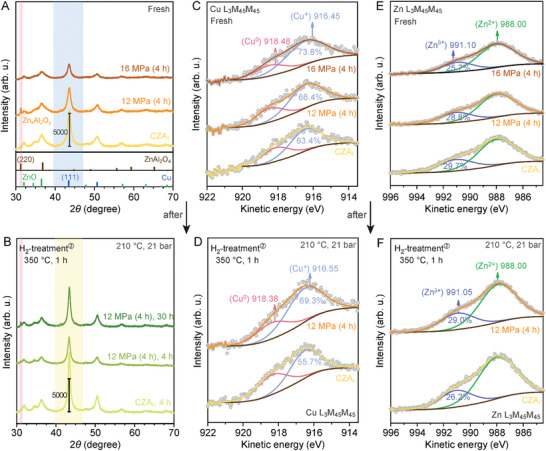
Structures of the fresh and spent catalysts at 210  °C and 21 bar. A,B) XRD patterns. C–F) Quasi in situ XPS Auger spectra of Cu LMM (C,D) and Zn LMM (E,F).

To find out the underlying structural explanation for the observed trend, we performed X‐ray diffraction (XRD) and quasi in situ X‐ray photoelectron spectroscopy (XPS) measurements on the fresh and activated catalysts. All catalysts show comparable specific surface areas (55 m^2^ g^−1^), pore characteristics (pore volume, 0.25–0.28 cm^3^ g^−1^; average pore diameter, 19.28–23.43 nm), Cu and Zn contents, indicating overall structural robustness (Table S2 and Figure S5, Supporting Information). A significant decrease in the diffraction peak intensity for the SC CO_2_‐activated samples reveals an increase in amorphization (**Figure** **2**A).^[^ ^12^ ^]^ Additionally, the average crystallite sizes of the metallic Cu^0^ nanoparticles, derived from the Cu (111) peak, were found to be 9.3 (CZA_r_), 8.7 (12 MPa), and 9.8 (16 MPa) nm, respectively, indicating pressure‐dependent grain sizes. The reduced grain size is related to the increased number of the Cu^0^–O–Zn*^δ^* ^+^ interface active sites, as confirmed by N_2_O reactive frontal chromatography (N_2_O‐RFC) measurements.^[^ ^13^ ^]^

Notably, a new peak at 31.2° appears on the 12 MPa (4 h) sample. It is associated with the (220) plane of ZnAl_2_O_4_. The formation of Zn,Al‐spinel in the CZA_r_ catalysts under reaction conditions usually involves the crystallization of amorphous Al‐oxide species and consumption of crystalline ZnO, which would lead to catalyst deactivation.^[^ ^14^ ^]^ In contrast, no diffraction peaks of Al‐oxide species were observed on our samples, and the crystallinity of ZnO decreased after activation, suggesting a different formation mechanism of the spinel phase in the SC CO_2_–EtOH system. Note that the formation of Zn*_x_* Al_2_O*_y_* can hinder the reduction of Zn*^δ^* ^+^ species and the sintering of Cu nanoparticles under long‐term reaction, thereby enhancing the stability of the activated catalysts (Figure 2B and Figure S6, Supporting Information).^[^ ^15^ ^]^ The decrease in the intensity of the diffraction peak for Zn*_x_* Al_2_O*_y_* is probably caused by reaction‐driven surface reconstruction.^[^ ^16^ ^]^ The XPS results of Zn 2p_3/2_ for the fresh and spent catalysts at 210 °C and 21 bar further reveal changes in the Zn coordination environment (Figure S7, Supporting Information).^[^ ^17^ ^]^

Quasi in situ XPS analysis provided deep insights into the near‐surface structure of the tested catalysts. The atomic ratio of Cu to Zn follows the order of 12 MPa (4 h) (0.83) > 16 MPa (4 h) (0.77) > CZA_r_ (0.75), indicating that the coverage of ZnO*_x_* on the Cu surface induced by SMSI is reduced after activation. This observation indicates that the optimal catalyst activity is not dominated by the Cu–ZnO*_x_* interfaces, which are recognized as the sole active sites for methanol synthesis.^[^ ^18^ ^]^ The Cu and Zn LMM Auger spectra (Figure 2C,E) show that the surface proportion of Cu^+^ species increases after further treatment in the SC CO_2_–EtOH system, whereas with a lower proportion of Zn*^δ^* ^+^ species.^[^ ^13^, ^19^ ^]^ Upon further exposure to H_2_ at 350 °C, a complete reduction of Cu occurred in CZA_r_, as evidenced by the disappearance of the Cu^+^ signal. Nevertheless, the Auger parameter of Cu for the activated catalysts differs from that of CZA_r_, evidencing a mixture of Cu^0^ and Cu^+^ (Figure S8, Supporting Information). More intriguingly, the amounts of Zn*^δ^* ^+^ in the activated catalysts increase significantly. Under reaction conditions at 210 °C (21 bar or atmospheric pressure), a further decrease in the atomic percentages of Cu^+^ and Zn*^δ^* ^+^ was observed on the CZA_r_ catalyst (Figure 2D,F and Figure S9, Supporting Information). However, the reaction mixture only slightly reduces the amount of Zn*^δ^* ^+^ in the activated samples, indicating a higher stability. The increased amount of Cu^+^ might result from the reformation of a slight overlayer.

**Figure 3:**
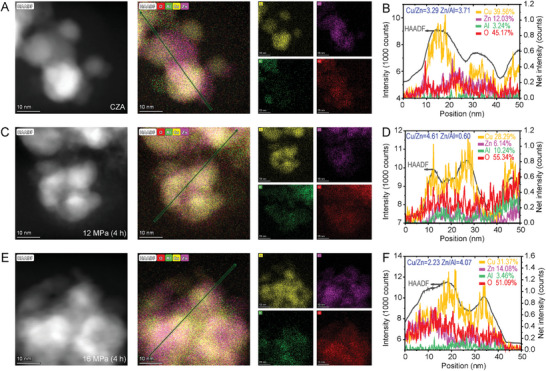
TEM characterization of the catalysts. A) HAADF STEM image and EDX elemental maps of CZA_r_. Yellow: Cu; pink: Zn; green: Al; red: O. B) HAADF intensity and EDX line profiles along the green line in A). C,D) For the 12 MPa (4 h) sample. E,F) For the 16 MPa (4 h) sample.

High‐angle annular dark‐field scanning transmission electron microscopy (HAADF STEM) imaging and energy‐dispersive X‐ray (EDX) mapping confirmed structural changes after the SC CO_2_ treatment. The HRTEM images show the apparent amorphization of Cu nanoparticles for the activated samples (Figures S10 and S11, Supporting Information). SC CO_2_‐induced amorphization has been demonstrated before, where metallic Cu° can be oxidized to amorphous Cu*_x_* O species with a high selectivity for C2+ products.^[^ ^20^ ^]^ Detailed analysis of the lattice spacing distribution suggested that ZnO overlayer on Cu nanoparticles with an uncontrollable SMSI phenomenon in the CZA_r_ catalyst, i.e., fully covered Cu nanoparticles, coexist with partially covered and partially uncovered ones (**Figures** **3**A and S12, Supporting Information). The EDX line scans across a particle in CZA_r_ show a Cu:Zn atomic ratio of 3.5 in the near‐surface region (Figure 3B), which is consistent with previously reported results. The Cu:Zn atomic ratio at the surface of the catalyst decreased after reducing H_2_ treatment.^[^ ^21^ ^]^ However, activating the catalyst with the SC CO_2_–EtOH system initiated clear reconstruction at the Cu–ZnO*_x_* interface. The ZnO overlayer vanished in 12 MPa (4 h) but became richer in the 16 MPa (4 h) sample (Figure 3C–F).

The retraction of overgrown ZnO on the surface of the 12 MPa (4 h) sample was also evidenced by an increased Cu:Zn atomic ratio of 4.61. In the high‐performance catalyst studied in our work, ZnO with a small particle size (<5 nm) is uniformly distributed around the Cu particles, contributing to the exposure of a large Cu surface area of up to ≈87.61 m^2^ g^−1^ (Table S2, Supporting Information), as well as to the optimization of the Cu–ZnO*_x_* interface. The N_2_O‐RFC data reveal that the Cu dispersion in the CZA_r_ catalyst, when activated with the SC CO_2_–EtOH mixture, is closely related to the treatment pressure. The dispersion follows the trend of 12 MPa (4 h) (12.95%) > 16 MPa (4 h) (10.40%) > CZA_r_ (10.08%) (Figure S13, Supporting Information). In addition, the enrichment of Al can be considered as being coupled to the formation of the new Zn*_x_* Al_2_O*_y_* phase. The beneficial role of structural reconstruction at the Cu–ZnO*_x_* interface was further confirmed by the almost unchanged crystallite sizes and amorphous structure over the spent 12 MPa (4 h) catalyst, although the SMSI‐induced partial coverage of the Cu surface with ZnO*_x_* seems to have occurred again (Figure S14, Supporting Information). Recuperation and repeated processing in the SC CO_2_–EtOH system were shown to facilitate these structural regenerations (Figure S15, Supporting Information).

### 2.3 Origin of the Active Sites

When CO_2_ fluid is subjected to temperatures above its critical point of 31.1 °C and pressures exceeding 7.38 MPa, it enters a SC state known as a SC CO_2_ fluid, in which chemical reactions and materials synthesis that cannot be achieved with traditional solvents can occur. To gain insights into the reactions in SC CO_2_, one should first know that the critical characteristics of SC CO_2_ are the inhomogeneity in space and the fluctuation in time.^[^ ^22^ ^]^ When a solute is immersed in such an inhomogeneous medium, the average solvent density in the local region around a solute molecule will essentially differ from the bulk density, with typical increases/decreases of 50–300%. In addition, by adjusting the relevant parameters of the system, one can adjust the lifetime of the solvation structure and, consequently, the fluctuation of the polar environment (correlation time) around the solute molecule. In this context, SC CO_2_ systems are being increasingly appreciated, especially very recently, for that the spatial inhomogeneity in the solvent density around reacting particles can dramatically affect the kinetics of diffusion‐controlled reactions.^[^ ^23^ ^]^

**Figure 4:**
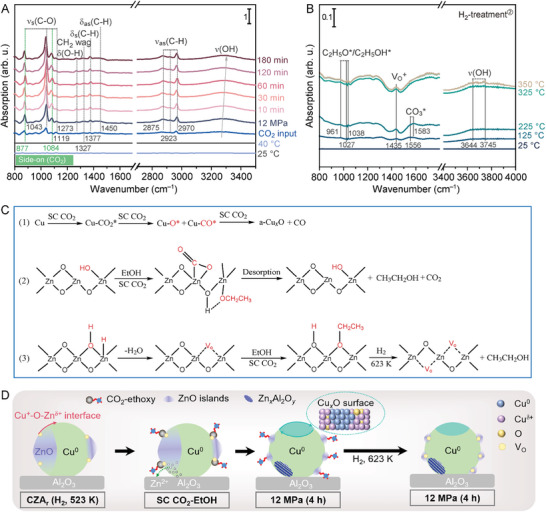
Structural evolution of the catalyst under the SC CO_2_ environment. A) Time‐dependent attenuated total reflectance infrared spectra of the fresh CZA_r_ in the SC CO_2_–EtOH solvent system. B) In situ temperature‐programmed DRIFTS of 12 MPa (4 h) with the spectra collected when the temperature was increased to 350 °C at a ramping rate of 5 °C min^−1^ in H_2_. C) Proposed reaction mechanism for SC CO_2_‐induced activation. The illustrations show how the SC CO_2_ and EtOH mixture critically affect the Cu/ZnO/Al_2_O_3_ structure. D) Schematic illustration of the structural reconstructions.

We employed attenuated total reflectance infrared spectroscopy connected with a SC CO_2_ apparatus to study the reaction kinetics dominated by fluctuation and to determine the origin of active sites in the SC CO_2_–EtOH system. **Figure** **4**A shows the time‐dependent spectra of the fresh CZA_r_ subjected at 40 °C and 12 MPa. After the introduction of CO_2_, a dynamic change in the adsorbates on the catalyst surface was observed. Ethoxy groups (^*^OC_2_H_5_) bound at “bridging oxygen” vacancies (Zn–O_br_–OC_2_H_5_) with strong, highly characteristic bands at 1043, 1377, 1450, and 2875–2970 cm^−1^ were identified as the main adsorbed species.^[^ ^24^ ^]^ The peak intensity of Zn–OC_2_H_5_ (1119 cm^−1^) on CZA_r_, where the ethoxy groups are bound to the surface Zn atoms, is much weaker than that of Zn–O_br_–OC_2_H_5_. The bands at 1273 and 1327 cm^−1^ can be assigned to the δ_OH_ and CH_2_ wagging modes of a small number of ethanol molecules physisorbed on the surface. Note that surface‐adsorbed ^*^C_2_H_5_OH is believed to poison active sites and prevent the migration of oxide species.^[^ ^10^, ^11^ ^]^ Notably, the migration and secondary uniform nucleation of ZnO*_x_* on the Cu surface is realized in the fluctuating SC CO_2_–EtOH medium. On the other hand, the broad bands at 3200–3400 cm^−1^ are derived from the intermolecular hydrogen bonds between CO_2_ and ethanol, which can significantly promote the polarity and solubility parameters of SC CO_2_.^[^ ^25^ ^]^

Meanwhile, the notable features of the C─O symmetric stretching modes at 877 and 1084 cm^−1^ suggest a η^2^(C, O) structural motif of metal–CO_2_ interactions, i.e., CO_2_ being fixed in a “side‐on” coordination manner.^[^ ^26^ ^]^ The side‐on bonded CO_2_ is known to be unstable as it is a weakly bound van der Waals‐type complex. The removal of CO_2_ may thus occur during decompression. These observations suggest that the surface‐adsorbed ^*^OC_2_H_5_ and CO_2_ can act as the driving force for the migration of Zn species. The in situ diffuse reflectance infrared Fourier transform spectroscopy (DRIFTS) of the CZA_r_ and 12 MPa (4 h) catalysts after H_2_ treatment reveals that the two samples have markedly different adsorption features (Figure 4B and Figure S16, Supporting Information). As the temperature was increased, adsorbed C_2_H_5_O/C_2_H_5_OH (961, 1027, and 1038 cm^−1^),^[^ ^27^ ^]^ water (3644 and 3745 cm^−1^), and surface carbonate species (1556 and 1583 cm^−1^) gradually appeared on the surface of the 12 MPa (4 h) catalyst.^[^ ^28^ ^]^ The emergence of carbonate vibrational bands, correlating with residual CO_2_ in the pipeline, underscores the high activity of ZnO*_x_* nanoparticles towards CO_2_ conversion. In particular, the band at 1435 cm^−1^ over the 12 MPa (4 h) catalyst that arises from V_O_^+^, where the oxygen vacancy (V_O_) adsorbs a H_2_ molecule and subsequently transfers electrons to it,^[^ ^29^ ^]^ shows noticeable enhancement compared with the CZA_r_ catalyst. Meanwhile, the core level fitting of the O 1s signal also shows an increased presence of V_O_ on the surface of the 12 MPa (4 h) catalyst after H_2_ treatment (Figure S17, Supporting Information). These results suggest that surface O_br_–OC_2_H_5_ groups are removed as ethanol gas by protonation, thereby enabling the 12 MPa (4 h) catalyst with increased V_O_.

It is worth summarizing the evidence for the participation of ethanol and CO_2_ adsorbates on the catalyst in reconstructing the Cu surface, ZnO*_x_* overlayer, and largely improved CO_2_ hydrogenation reactivity (Figure 4C). For the Cu surface, the dissociation of CO_2_ molecules generates *CO and *O species on the Cu surface. The subsequent penetration of *O inside the Cu lattice transforms metallic Cu^0^ into an amorphous Cu*_x_* O shell,^[^ ^20^ ^]^ which generates a large number of Cu^0^–Cu^+^ surface sites. Based on the TEM and IR results in Figures 3 and 4, ethoxy groups and CO_2_ molecules are responsible for the migration of Zn species, as discussed in other works.^[^ ^24^, ^30^ ^]^ We emphasize that ethanol species interact with Zn species in two different ways to reconstruct the ZnO*_x_* overlayer. One is the Zn–OC_2_H_5_ species, which can readily be removed through combination with surface hydroxyl groups to desorb. The other is the O_br_–OC_2_H_5_ species, which is bridge‐bonded to two subsurface Zn cations and less active. Its removal occurs in the high‐temperature range. On the other hand, the unreactive ethoxy groups can also be removed by protonation under reducing H_2_ conditions, creating more V_O_ in the activated catalyst. Such optimization of the Cu–ZnO*_x_* interface structure in the SC CO_2_–EtOH solvent system can significantly increase the number of the Cu^0^–O–Zn*^δ^* ^+^ interface active sites. In addition, we hypothesize that the interaction of CO_2_ with Zn species can induce the incorporation of Zn in the amorphous Al_2_O_3_ support and that the spinel‐like structure of γ‐Al_2_O_3_ can facilitate the formation of the new Zn*_x_* Al_2_O*_y_* phase.^[^ ^31^ ^]^ An additional shoulder peak centered at 70 ppm (Al_IV_ site) in the ^27^Al magic‐angle spinning nuclear magnetic resonance and a simultaneous redshift in the Al_VI_ peak frequency strongly support our hypothesis (Figure S18, Supporting Information).^[^ ^15a^ ^]^ This new phase helps to stabilize Zn*^δ^* ^+^ species under the reaction conditions, thereby enhancing the catalytic activity of the Cu^0^–O–Zn*^δ^* ^+^ interface active sites. Based on the above results, we propose the mechanism for adsorbate (ethanol and SC CO_2_ molecule)‐induced activation and characteristics (Figure 4D). The behavior of the Cu/ZnO/Al_2_O_3_ surface depends on the reaction mixture and conditions, and the critical keys to high performance are the large accessible activated Cu*_x_* O surface area and optimized Cu–ZnO*_x_* interface.

### 2.4 Analysis of the Reaction Pathways

Temperature‐programmed desorption (TPD) of CO_2_, CO, H_2_, and NH_3_ experiments were carried out to examine the effect of the activated Cu*_x_* O surface and optimized Cu–ZnO*_x_* interface on the species adsorbed on the catalyst surface under the reaction conditions.

**Figure 5:**
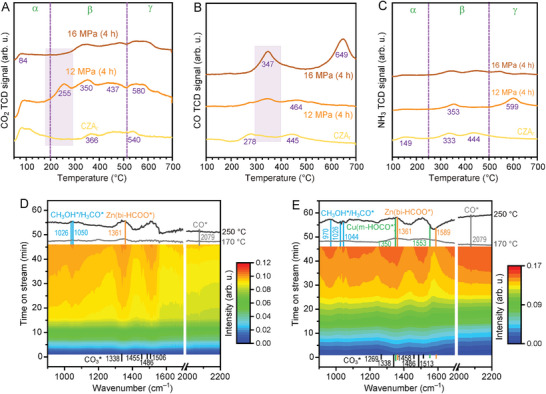
Identification of the active sites. A) CO_2_‐TPD, B) CO‐TPD, and C) NH_3_‐TPD curves of the CZA_r_, 12 MPa (4 h), and 16 MPa (4 h) catalysts. D, E) Time‐dependent in situ DRIFTS spectra of the CZA_r_ (D) and 12 MPa (4 h) (E) catalysts under feed gas with an H_2_/CO_2_ molar ratio of 3:1 from 170 to 210 °C under atmospheric pressure.

The CO_2_‐TPD results of all the catalysts confirm the presence of physically adsorbed CO_2_ on hydroxyl groups (α < 150 °C), chemically adsorbed CO_2_ on the metal oxide (200 < β < 500 °C), and Cu–ZnO*_x_* interfacial sites (γ > 500 °C) (**Figure** **5**A).^[^ ^32^ ^]^ Interestingly, the activated catalysts have distinct β and γ peaks compared with CZA_r_, revealing a synchronous enhancement of surface and interface active sites. Particularly, an additional β peak at 255 °C on the 12 MPa (4 h) catalyst suggests a positive effect of the Cu*_x_* O surface on the CO_2_ adsorption. H_2_‐TPD data (Figure S19, Supporting Information) further demonstrate that the enhanced active sites endow the 12 MPa (4 h) catalyst with the strongest H_2_ adsorption/activation capacity (0.558 mmol g^−1^).^[^ ^33^ ^]^ Meanwhile, the CO‐TPD profile of the 12 MPa (4 h) catalyst exhibits a lower overall CO desorption and higher desorption temperature, revealing that SC CO_2_ activation can efficiently adsorb and transform *CO intermediates (Figure 5B).^[^ ^34^ ^]^ An increased acid site was also observed on the 12 MPa (4 h) catalyst (Figure 5C), which might be caused by the incorporation of Zn in γ‐Al_2_O_3_.^[^ ^35^ ^]^ Note that the changes in acid sites for the 12 MPa (4 h) catalyst are beneficial for high selectivity to CH_3_OH even at a higher conversion rate. These findings demonstrate that exposing the CZA_r_ catalyst to the SC CO_2_–EtOH mixture under reasonable pressure during activation can increase the number of the Cu^0^–O–Zn*^δ^* ^+^ interface sites owing to the secondary nucleation and growth of ZnO, as well as generate a massive number of the Cu^0^–Cu^+^ surface sites through the formation of a significantly accessible activated Cu*_x_* O amorphous shell. In contrast, excessive treatment results in a thick ZnO*_x_* overlayer, reducing the number of accessible Cu^0^–Cu^+^ surface sites on the surface of Cu*_x_* O.

To date, the formate mechanism, where CO_2_ binds through a C atom and a H atom coming from the dissociation of H_2_ on metallic Cu^0^, forming a bidentate formate (bi‐HCOO*) intermediate on active ZnO sites, is most commonly identified as the reaction mechanism of CO_2_ hydrogenation to CH_3_OH.^[^ ^4b^ ^]^ Another pathway involves CO generated from the RWGS reaction through HOCO* species. It is generally accepted for the hydrogenation of CO_2_ on a Cu catalyst with inert support.^[^ ^36^ ^]^ Analysis of the structural evolution under the SC CO_2_ conditions and the catalytic performance over the activated catalysts have shown the favorable roles of the newly created Cu^0^–Cu^+^ surface sites and optimal Cu^0^–O–Zn*^δ^* ^+^ interface sites in CO_2_ hydrogenation to CH_3_OH. We thus hypothesize that both the formate (HCOO*) and CO‐involved mechanisms act simultaneously over the activated catalyst during realistic conditions.

In situ temperature‐programmed DRIFTS studies at atmospheric pressure were undertaken to gain further insight into the reaction pathway over the activated catalyst (170–250 °C). The catalysts were first reduced with a stream of H_2_ at 350 °C for 1 h and then rinsed with Ar to remove weakly adsorbed molecules. Under the reaction conditions, the DRIFT spectra of the catalysts (3H_2_:CO_2_ treatment) show similar CO_3_* species (Cu–CO_3_* at 1269 and 1338 cm^−1^, Zn–CO_3_* at 1455–1513 cm^−1^) and the adsorption of formate on Zn (bi‐HCOO* at 1361 and 1589 cm^−1^) with two O atoms bound to two adjacent Zn atoms (Figure 5D,E).^[^ ^18^ ^]^ The peaks at 1026–1050 cm^−1^ can be assigned to the CH_3_OH* and Zn–H_3_CO* species. Overall, the generation and consumption of Zn–HCOO* on the 12 MPa (4 h) catalyst were extremely fast compared to those of CZA_r_, even at the very early stage (170 °C), demonstrating a highly reactive reaction on the activated catalyst. Furthermore, the single vibrational peak of Zn(bi‐HCOO*) on CZA_r_ suggests that the Cu^0^–O–Zn*^δ^* ^+^ interface site is the primary active site for CO_2_ activation and conversion. Noticeably, except for the common formate species, adsorbed Cu–H_3_CO* (970 cm^−1^) and the formation of monodentate HOCO* species (m‐HOCO* at 1350 and 1553 cm^−1^) on Cu with the C atom bonded to the active Cu site appear on the activated catalyst.^[^ ^37^ ^]^ As the temperature was increased, the conversion of m‐HOCO* to H_3_CO* species was much faster over bi‐HCOO* on the 12 MPa (4 h) catalyst. More importantly, the DRIFT spectra of the activated catalyst show a twofold decrease in the integrated area of CO species (2079 cm^−1^) compared to CZA_r_, especially at a higher temperature (250 °C), indicating the efficient hydrogenation conversion of *CO intermediates on the 12 MPa (4 h) catalyst. The identification of Zn(bi‐HCOO*) and Cu(m‐HOCO*) intermediates and the decrease in the CO intensities, combined with the promotion of the reactivity of the activated catalyst, demonstrate that the exceptional reactivity induced by the SC CO_2_–EtOH treatment is mediated by the dual‐response pathway involving the formate and RWGS + CO hydrogenation mechanisms.

Accordingly, all components in the activated catalyst play important roles in the reaction. The involved components and roles can be summarized as follows. (i) The metallic Cu^0^ sites at the surface and interface dominate the adsorption and activation of H_2_. (ii) The small ZnO species that are uniformly distributed around the Cu particles help the dispersion of the Cu phase and stabilize the active Cu^+^ sites at the surface of the Cu*_x_* O amorphous shell or the Cu–ZnO*_x_* interface. At the same time, the improved Cu–Zn synergy provides sufficient and stable Cu^0^–O–Zn*^δ^* ^+^ interface sites, which, together with the Cu^0^–Cu^+^ surface sites, activate CO_2_ and initiate the subsequent hydrogenation steps by different mechanisms. (iii) The formation of Zn*_x_* Al_2_O*_y_* and the changes in acidic sites caused by the incorporation of Zn in the amorphous Al_2_O_3_ support promote the stability and selectivity of the activated catalyst. In addition, we conducted a comparative experiment using a Cu/ZnO/SiO_2_ catalyst without Lewis acidic sites to further highlight the universality of the proposed supercritical CO_2_ activation strategy (Figure S20, Supporting Information).^[^ ^33^, ^38^ ^]^ Similar phenomena of the amorphization of the Cu component as well as the secondary nucleation and growth of ZnO were observed in the activated Cu/ZnO/SiO_2_ catalyst. As a result, the MeOH STY of the 12 MPa (4 h) Cu/ZnO/SiO_2_ sample increased approximately by two folds, with a higher selectivity (42%), in comparison with its pristine counterpart at the reaction temperature of 250 °C.

Density functional theory (DFT) calculations provided additional fundamental insights into the MeOH reaction pathway over the activated catalyst. We note that even theoretically, it is challenging to elucidate such a promotion effect at the atomic level.^[^ ^4b^ ^]^ We therefore proposed a simplified alternative solution, which is to calculate the adsorption energies of CO_2_ and the key intermediates on the different sites. Cu nanoparticles were represented by a Cu(111) surface, which dominates on the X‐ray diffraction patterns. The Cu*_x_* O and Cu–ZnO*_x_* sites under the reaction conditions were modeled using the optimized ZnO/Cu(111) structure, where a number of O atoms bind to Cu atoms on the surface (Figure S21, Supporting Information).

**Figure 6:**
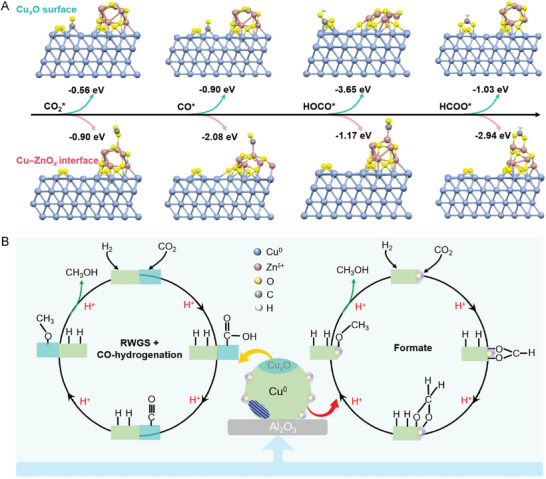
Analysis of the reaction pathway. A) DFT‐optimized geometries of CO_2_ and key intermediates adsorbed on the different sites of the activated catalyst and their adsorption energies, where the upper ones belong to Cu*_x_* O and the bottom ones belong to Cu–ZnO*_x_* on the 12 MPa (4 h) catalyst. Cu: blue; Zn: pink; O: yellow; C: grey; and H: white. B) Schematic diagram of the parallel reaction mechanisms on the Cu*_x_* O surface and Cu–ZnO*_x_* interface of the 12 MPa (4 h) catalyst for methanol synthesis from CO_2_ hydrogenation.

**Figures** **6**A and S22 (Supporting Information) show the DFT‐optimized geometries of CO_2_ and the key intermediates adsorbed on the Cu*_x_* O surface and Cu–ZnO*_x_* interface, respectively. The corresponding adsorption energies on the different sites were calculated (Table S3, Supporting Information). We can see that the Cu*_x_* O surface that arises from metallic Cu^0^ allows the binding of CO_2_ and CO, which can efficiently activate CO_2_ and suppress CO desorption. Furthermore, the HOCO* adsorption energy over the Cu*_x_* O surface is higher than that on the Cu–ZnO*_x_* interface, indicating that the RWGS + CO hydrogenation route can be enhanced over the Cu*_x_* O surface. In contrast, the preferential adsorption of the HCOO* intermediate at the Cu–ZnO*_x_* interface facilitates formate hydrogenation. These results further support the hypothesis that both the surface Cu^0^–Cu^+^ sites and the interfacial Cu^0^–O–Zn*^δ^* ^+^ sites are active for MeOH synthesis from the CO_2_–H_2_ reaction, which involves different elementary reaction processes. Figure 6B illustrates the dual‐response pathway of CO_2_ hydrogenation to MeOH, where the Cu^0^ site is responsible for H_2_ activation, while the Cu^+^ and Zn*^δ^* ^+^ sites are proposed to promote not only CO_2_ activation but also its further hydrogenation. The initial step of CO_2_ hydrogenation on the Cu^0^–O–Zn*^δ^* ^+^ interface sites occurs through a formate mechanism, where CO_2_ reacts with a surface H* to generate an adsorbed bi‐HCOO* species. This bi‐HCOO* species is then further hydrogenated, forming a H_2_COO* species, which undergoes sequential hydrogenation at the Cu–ZnO*_x_* interface to yield H_3_CO* as the reactive intermediate for MeOH synthesis. The Cu–CO_3_* species are easily desorbed to produce byproduct CO in this pathway. The parallel RWGS + CO hydrogenation mechanism occurs on the Cu^0^–Cu^+^ surface sites, which is preferred at higher reaction temperatures. The adsorbed CO_2_ is hydrogenated to form an m‐HOCO* species through Cu^+^, followed by further hydrogenation that splits into CO* and H_2_O. The further hydrogenation reaction of CO* gives a H_3_CO* intermediate, with the final formation of CH_3_OH. The synergistic effect of the Cu–ZnO*_x_* interface and Cu*_x_* O surface enables the Cu/ZnO/Al_2_O_3_ catalyst to be highly active, selective, and stable.

## 3 Conclusion

In summary, we have presented a supercritical CO_2_ activation strategy that synchronously perfects the SMSI‐induced Cu^0^–O–Zn*^δ^* ^+^ interface and exposed Cu^0^–Cu^+^ surface sites by manipulating the adsorbate diffusion kinetics. Adsorbed *OC_2_H_5_ and CO_2_ species on ZnO*_x_* have been demonstrated to effectively homogenize the secondary nucleation sites of ZnO*_x_* on the Cu surface and cause the incorporation of Zn in the Al_2_O_3_ support. Further removal of *OC_2_H_5_ creates more V_O_ in the activated catalyst, and the formation of the Zn*_x_* Al_2_O*_y_* spinel phase contributes to the improved stability of Zn*^δ^* ^+^ species under the reaction conditions. Moreover, CO_2_ adsorption on exposed metallic Cu^0^ results in the formation of an activated Cu*_x_* O amorphous shell. Such a structural evolution, including an enlarged accessible Cu*_x_* O surface and optimized Cu–ZnO*_x_* interface, unlocks the dual‐response pathway in methanol synthesis. CO_2_ preferentially adsorbs on the Cu*_x_* O surface in a monodentate bonding mode and undergoes hydrogenation through the reverse water‐gas shift plus CO hydrogenation pathway. At the same time, the formate pathway occurs on the Cu–ZnO*_x_* interface. The opportunities offered by our strategy to design active surface and interface sites at the atomic scale open new avenues for improving the reactivity of supported metal catalysts.

## Conflict of Interest

The authors declare no conflict of interest.

## Author Contributions

Y.N.Z., S.R.Z., and J.F.W. conceived the project. Y.N.Z., J.Y.J., S.R.Z., and J.F.W. supervised the project. Y.N.Z., Y.S.W., and R.J.L. performed the experiments, collected, and analyzed the data. Y.N.Z. and J.F.W. drafted the manuscript and figures, and all authors revised them.

## Supporting information



Supporting Information

## Data Availability

The data that support the findings of this study are available from the corresponding author upon reasonable request.
