# Supplementary material for: Supercritical CO2 Activation Enables an Exceptional Methanol Synthesis Activity Over the Industrial Cu/ZnO/Al2O3 Catalyst
Source: Adv Sci (Weinh). 2025 Mar 7;12(17):2500118. doi: 10.1002/advs.202500118 (PMC12061296; doi:10.1002/advs.202500118)
Supplement: Supplementary file 1 — Supporting Information [file ADVS-12-2500118-s001.docx]

Supporting Information

Supercritical CO_2_ Activation Enables an Exceptional Methanol Synthesis Activity over the Industrial Cu/ZnO/Al_2_O_3_ Catalyst

Yannan Zhou, Jingyun Jiang,* Yushun Wang, Ruijie Liu, Shouren Zhang,* and Jianfang Wang*

Dr. Y. N. Zhou, R. J. Liu, Prof. S. R. Zhang
Henan Provincial Key Laboratory of Nanocomposites and Applications
Institute of Nanostructured Functional Materials
Huanghe Science and Technology College
Zhengzhou, Henan 450006, China
E-mail: shourenzhang@infm.hhstu.edu.cn

Prof. J. F. Wang
Department of Physics
The Chinese University of Hong Kong
Shatin, Hong Kong SAR 999077, China
E-mail: jfwang@phy.cuhk.edu.hk

Dr. J. Y. Jiang
College of Materials Science and Engineering
Zhengzhou University
Zhengzhou, Henan 450052, China
E-mail: jiangjingyun@zzu.edu.cn

Y. S. Wang
School of Chemistry and Chemical Engineering
Henan University of Science and Technology
Luoyang, Henan 471023, China

**Contents**

1. Experimental Section

1.1. Computational Details

1.2. Chemicals and Materials

1.3. Catalyst Synthesis

1.4. Catalyst Characterization

1.5. CO_2_ Hydrogenation

1.6. Statistical Analysis

2. Supporting Figures

3. Supporting Tables

4. Supporting References

**1. Experimental Section**

**1.1. Computational Details**

All first-principles calculations in this work were performed using the density functional theory (DFT) as implemented in the Vienna *Ab initio* Simulation Package (VASP), which has been extensively employed to explore the structures and properties of materials. The generalized-gradient approximation (GGA) of Perdew-Burke-Ernzerhof (PBE) was used for the electronic exchange-correlation function. Projected augmented wave (PAW) potentials were employed to characterize ionic cores and to account for valence electrons, with a plane wave basis set and a kinetic energy cutoff of 450 eV. The PBE-based DFT-D3 correction was considered to describe van der Waals interaction. Geometry improvements were made with a force convergence of less than 0.05 eV Å^−1^. Monkhorst-Pack *k*-points of 2 × 3 × 1 were used for all calculations. In all calculations, the two atomic layers at the bottom of Cu(111) were assumed to remain fixed, and the adsorption energy (*E*_a_) was calculated using according to *E*_a_ = *E*(total) − *E*(slab) − *E*(reference).

**1.2. Chemicals and Materials**

Ethanol (C_2_H_5_OH, 99.99%, Aladdin, China) and acetone (C_3_H_6_O, 99.99%, Aladdin, China) were of analytical grade and used without further purification. The industrial CuO/ZnO/Al_2_O_3_ catalyst was purchased from Sichuan Shutai Chemical Technology. CO_2_ with a purity of 99.99% was purchased from Zhengzhou Shuangyang Gas Co. Deionized water was prepared from doubly distilled water.

**1.3. Catalyst Synthesis**

*Reducing the Industrial CuO/ZnO/Al_2_O_3_ Catalyst.* The industrial CuO/ZnO/Al_2_O_3_ catalyst (CZA) was first ultrasonically cleaned with acetone and ethanol to remove impurities, followed by a temperature-programmed reduction (TPR) process in a 10 vol%H_2_/N_2_ flow within the range of 30–250 °C at a heating rate of 5 °C min^−1^. The final reduction product was denoted as CZA_r_.

*Preparation of Cu/ZnO/SiO_2_ Catalyst*. 3.0 g of SiO_2_ support was vacuum dried at 110 °C for 1 h to eliminate gas impurities from the SiO_2_ pores. The prepared SiO_2_ was then dispersed in 200 g of deionized water, designated as suspension A. Subsequently, 1.863 g of Cu(NO_3_)_2_·3H_2_O and 1.236 g of Zn(NO_3_)_2_·6H_2_O were dissolved in deionized water to create solution B. At the same time, a 1 M Na_2_CO_3_ solution was prepared, referred to as solution C. Solution C was mixed with suspension A and stirred at 70 °C for 10 minutes to form suspension D. Solution B was then rapidly added to suspension D, and using solution C to adjust its pH to 7. Following an aging process at 70 °C for 1 h, the precursor was collected by hot filtration, washed with deionized water three times, and vacuum dried at 60 °C for 12 h. Finally, the as-obtained precursor was calcined at 350 °C for 4 h, yielding a green sample powder designated as CZS. Before the hydrogenation reaction, CZS was pre-reduced in a 10 vol%H_2_/N_2_ flow (60 mL min^−1^), denoted as CZS_r_.

*Activation of the Industrial CZA_r_ Catalyst.* The CZA_r_ powder (100 mg) was dispersed in ethanol (30 mL) and subjected to ultrasonic treatment for 30 min. The ultrasonicated CZA_r_ suspension was directly transferred into a supercritical CO_2_ apparatus composed mainly of a stainless steel autoclave with a heating jacket and a temperature controller. After the autoclave was heated to a designated temperature (40 °C), CO_2_ was charged into the reactor to the desired pressures (12 or 16 MPa) and held for a certain duration (2, 4, or 6 h) under continuous stirring. After CO_2_ was slowly released, the sediment was collected by centrifugation at 8,000 rpm for 10 min. The treated catalysts are denoted as *X* MPa (*Y* h), where *X* MPa and *Y* h represent the treatment pressure and time in the SC CO_2_–EtOH system, respectively.

**1.4. Catalyst Characterization**

The powder X-ray diffraction (XRD) patterns of all samples were collected at room temperature on a Bruker D8 Advance diffractometer using a germanium monochromator (Cu Kα radiation, 40 kV and 40 mA). The XRD measurements were carried out at a scan rate of 1 ° min^−1^.

Quasi *in situ* X-ray photoelectron spectra (XPS) and X-ray excited Auger spectroscopy data were obtained with a PHI VersaProbe 4 system equipped with an Al Kα X-ray source. A glovebox was used for the catalyst pretreatment, and the sample kept in N_2_ was directly transferred into the vacuum analysis chamber to avoid exposure to air.

TEM images and high-angle annular dark-field scanning transmission electron microscopy (HAADF-STEM) images were recorded on JEOL ARM-200F operated at an acceleration voltage of 200 kV. The elemental distributions of the samples were detected using the equipped energy-dispersive X-ray (EDX) analysis system for elemental mapping and linear scanning.

The Cu and Zn contents were determined using inductively coupled plasma optical emission spectrometry (ICP-OES) on an Agilent 7800 system.

For the Cu dispersion analysis, the samples were first reduced to Cu metal in a gas flow mixture of 10 vol% H_2_/Ar with the temperature ramped to 300 °C at 10 °C min^−1^ during the first H_2_-TPR treatment. After cooling to 50 °C, the chamber was purged with Ar for 60 min. The oxidation of Cu to Cu_2_O was realized by 10 vol% N_2_O/Ar treatment at 50 °C. A second H_2_-TPR treatment on the formed Cu_2_O was then conducted, following the same procedure as the first H_2_-TPR. The Cu dispersion was derived from the H_2_ uptake (*A*) in the two TPR processes: Cu dispersion = $\frac{2A_{2}(2nd TPR)}{A_{1}(1st TPR)}\times100\boldsymbol{\%}$. The exposed Cu surface area (*S*_Cu_) was calculated according to *S*_Cu_ (m^2^ g^−1^) = (2*A*_2_ × *N*_A_)/(*A*_1_ × *M*_Cu_ × 1.4 × 10^19^ × *W*_Cu_), where *N*_A_ is Avogadro’s number, *M*_Cu_ is the molar mass of Cu, *W*_Cu_ is the content of Cu, and 1.4 × 10^19^ is the number of Cu atoms per square meter on average.

The N_2_ adsorption-desorption isotherms of the samples were measured at −196 °C on a BELSORP-MAX apparatus. The samples were outgassed under vacuum at 200 °C for 3 h before N_2_ adsorption. The total pore volumes, average pore diameters, and pore size distributions were obtained from the N_2_ adsorption branches of the isotherms using the Barret-Joyner-Halenda method.

CO_2_ temperature-programmed desorption (CO_2_-TPD) was performed on a Micromeritics AutoChemII 2920 instrument. Specifically, the sample (50 mg) was first reduced in H_2_ (30 mL min^−1^) at 250 °C for 1 h. The gas was next switched to Ar flow (30 mL min^−1^) at the same temperature for 1 h. After the sample cooled down to 50 °C, the gas was switched to 10 vol%CO_2_/Ar flow (30 mL min^−1^) for 1 h, and the sample was further purged with Ar (30 mL min^−1^ for 30 min) to remove physically adsorbed CO_2_. CO_2_-TPD was finally performed by heating the sample from 50 °C to 700 °C with a heating rate of 10 °C min^−1^ in Ar. CO-TPD and H_2_-TPD were carried out using a similar procedure, except that 10 vol%CO/Ar or 10 vol%H_2_/Ar was used as the adsorption gas, respectively.

NH_3_-TPD experiments were performed on the Microtrac BELCAT II chemisorption analyzer. For NH_3_-TPD measurements, the sample (100 mg) was pre-treated in a quartz reactor with a purge of high-purity He at 100 °C for 60 min. NH_3_ adsorption was performed at 100 °C in an NH_3_/He mixture (10 vol% NH_3_) for 60 min, followed by TPD in He atmosphere by raising the temperature to 700 °C at a ramp rate of 10 °C min^−1^.

The ^27^Al NMR spectra were *ex situ* measured using a 3.2 mm MAS probe at a sample spinning rate of 20 kHz on a Bruker AVANCE III 500 MHz spectrometer. An Al(NO_3_)_3_ solution (1 M) was used as an external reference.

*Time-Dependent ATR-IR Spectra.* The supercritical CO_2_ apparatus for *in situ* attenuated total reflection infrared (ATR-IR) spectroscopy is mainly composed of a stainless steel autoclave with a heating jacket, a temperature controller, and an attenuated total reflection mid-infrared hollow optical fiber. Frourier transform infrared (FT-IR) spectra were recorded on an INVENIO R FT-IR spectrometer (Bruker, USA). A background spectrum was first collected in clean air in a typical *in situ* ATR-IR experiment. A suspension containing the CZA_r_ catalyst (100 mg) and ethanol (30 mL) was subsequently transferred into a 50 mL quartz container. The quartz container was placed in the supercritical CO_2_ apparatus and heated to 40 °C. When the temperature reached a steady value, CO_2_ was charged into the reactor to a pressure of 12 MPa and maintained for 3 h under continuous stirring. During this process, time-dependent spectra were continuously recorded. Typically, 16 scans were collected for each spectrum at a resolution of 4 cm^−1^.

*In situ Diffuse Reflectance Infrared Fourier Transform Spectroscopy (DRIFTS) Experiments.* An *in situ* Fourier transform infrared spectrometer (INVENIO S, Bruker, USA) equipped with a diffuse reflection cell (Harrick Praying Mantis) was used for *in situ* DRIFTS analysis. Spectra were collected at a resolution of 4 cm^–1^. Each spectrum was the average of 32 scans. About 20 mg of the catalyst was placed into a customized reaction cell. Before each measurement, the reaction cell was degassed with an ultrahigh vacuum pump and then purged with a dry Ar atmosphere for 30 min to sweep the impurities. The sample was first reduced with pure H_2_ within the range of 30–350 °C at a heating rate of 10 °C min^−1^ and held at 350 °C for 60 min. At the same time, the spectra were collected every 10 minutes. The reactor was next cooled to 170 °C, and the gas was switched to Ar flow at 30 mL min^−1^ for 60 min. The background was then collected. For *in situ* temperature-programmed DRIFTS, 20 mL min^–1^ of CO_2_/H_2_ (1:3 molar ratio) was fed to the cell. The sample was then heated to 250 °C at a rate of 5 °C min^−1^ in flowing CO_2_/H_2_, with the spectra collected every 2 minutes.

**1.5. CO_2_ Hydrogenation**

The CO_2_ hydrogenation experiments were performed using a Harrick flow reactor at varied pressures (**Figure S1**). The reactor temperature was controlled by a thermocouple and heating system connected to the Harrick reactor. Prior to the reaction, the catalyst (20 mg) was reduced *in situ* at 350 °C for 1 h under a 10 mL min^−1^ H_2_ flow. Subsequently, CO_2_ hydrogenation from 170 to 250 °C was performed with H_2_ + CO_2_ feedstock at a molar ratio of H_2_:CO_2_ = 3:1 and a flow rate of 20 mL min^−1^. The gas samples were collected after >2 h at each temperature step to allow the reaction to reach a steady state. The samples from the reactor outlet were injected into a gas chromatograph (GC) equipped with a thermal conductivity detector (TCD), a flame ionization detector (FID), and a mass spectrometer to help to identify the products. The TCD equipped with a TDX-01 column was used to analyze CO, CH_4_, and CO_2_, while the FID equipped with a SE-54 column was employed for CH_3_OH analysis. The stainless-steel gas line between the reactor and GC system was heated at 120 °C to avoid the condensation of the liquid products. Only CO and CH_3_OH were detected as the products over the tested catalysts. All the gas and products, including CO, CO_2_, and CH_3_OH, were calibrated using the standard gases to determine the peak positions, calibration factors, and retention time values. The calculation details of the CO_2_ conversion, product selectivity, and space-time yield (STY) are given as follows.

The conversion of CO_2_ (*X*_CO2_) was calculated according to $X\left( CO_{2} \right)=\frac{\left[ CO_{2} \right]_{\mathrm{in}}-\left[ CO_{2} \right]_{\mathrm{out}}}{\left[ CO_{2} \right]_{\mathrm{in}}}\times100\%$, where [CO_2_]_in_ and [CO_2_]_out_ represent the molar concentrations of CO_2_ in the inlet and outlet gas, respectively. The MeOH and CO STYs were calculated according to $\mathrm{MeOH}\left( or CO \right)\mathrm{STY}=\frac{n_{\mathrm{MeOH}}\left( \mathrm{or} n_{\mathrm{CO}} \right)}{m_{\mathrm{catalyst}}}\times60\left( \mathrm{mmol} g_{\mathrm{cat}}^{-1} h^{-1} \right)$, where $n=\frac{F\times\left[ \right]}{22.4}$ and *m*_catalyst_ is the mass of the catalyst. The symbol 🗆 represents MeOH or CO, *F* denotes the gas flow rate (mL min^−1^), and [🗆] represents the concentration (vol%) of the particular substance detected by online GC. The MeOH selectivity was determined according to $S\left( \mathrm{MeOH} \right)=\frac{MeOH STY}{MeOH STY+CO STY}$.

**1.6. Statistical Analysis**

Results are expressed as mean ± SEM (*n* = 3). For normally distributed data sets with equal variances, one-way ANOVA testing followed by a Tukey post-hoc test was carried out across groups. In all cases, significance was defined as *p* < 0.05. Statistical analysis was carried out using Python Software.

**2. Supporting Figures**


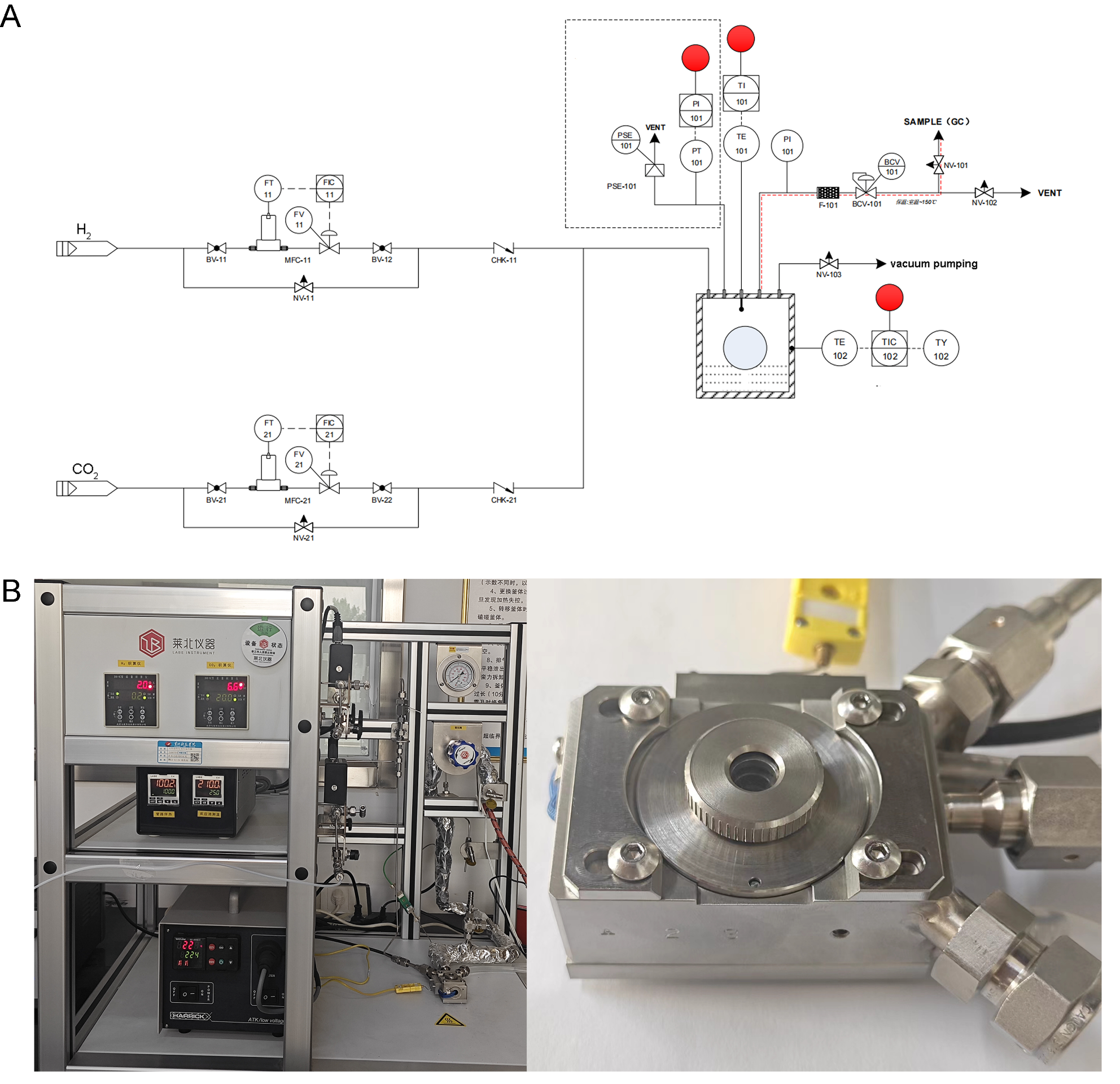


**Figure S1**. Flow reactor. A) Flow chart of the performance study system. B) Photograph of the reactor installation. The flow reactor was configured from a high-pressure Harrick reactor (HVC-MRA-5, Harrick Technology) with a heating cartridge and a K-type thermocouple.


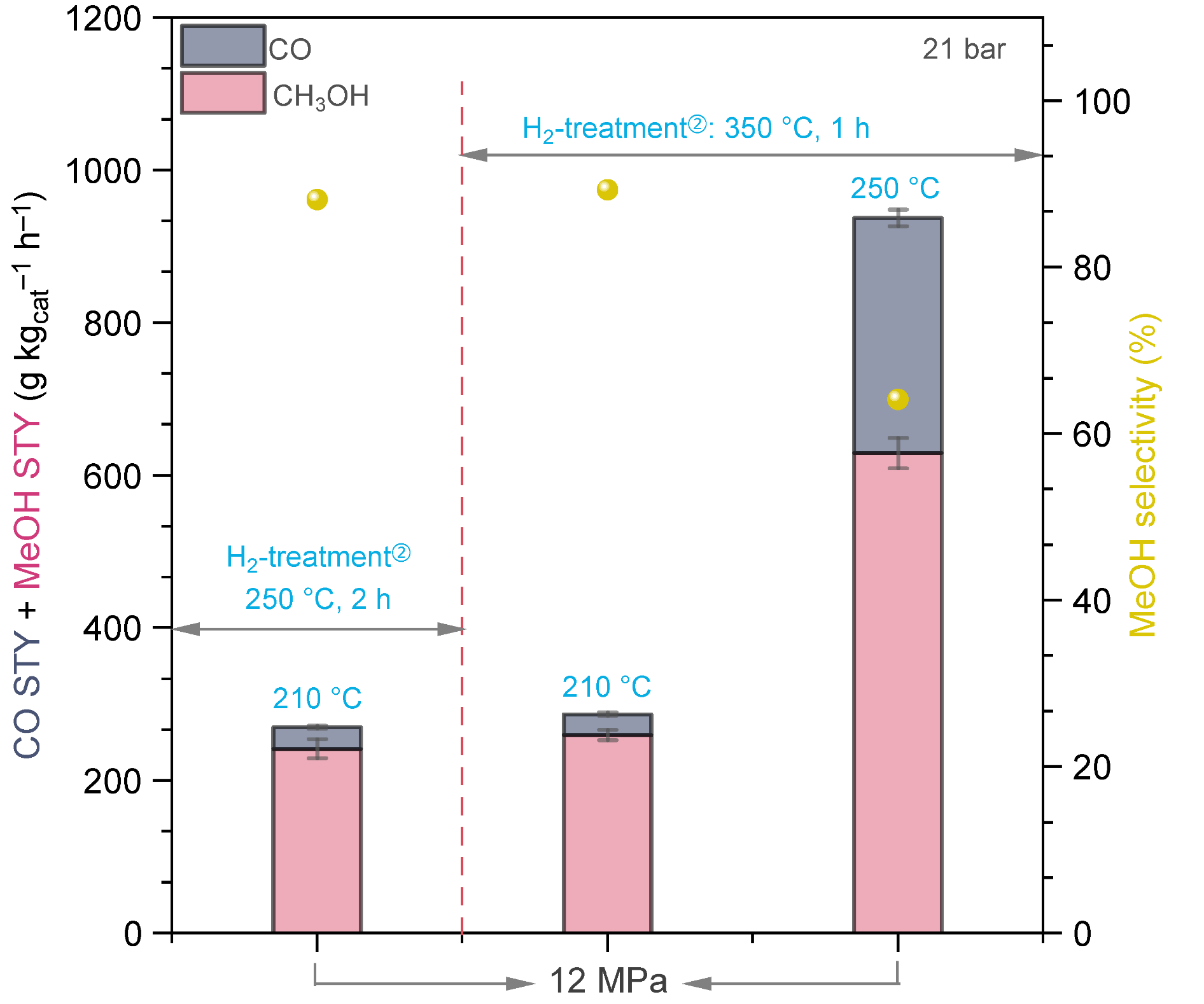


**Figure S2.** Catalytic performances of the 12 MPa (4 h) catalyst in CO_2_ hydrogenation to methanol. Changing the reducing gas condition (250 °C, 2 h) had a negligible effect on the activity of the catalyst, and the MeOH STY increased from 259.09 to 636.50 g kg_cat_^−1^ h^−1^ as the temperature was ramped to 250 °C. Results are expressed as mean ± SEM (*n* = 3), and significance was defined as *p* < 0.05.


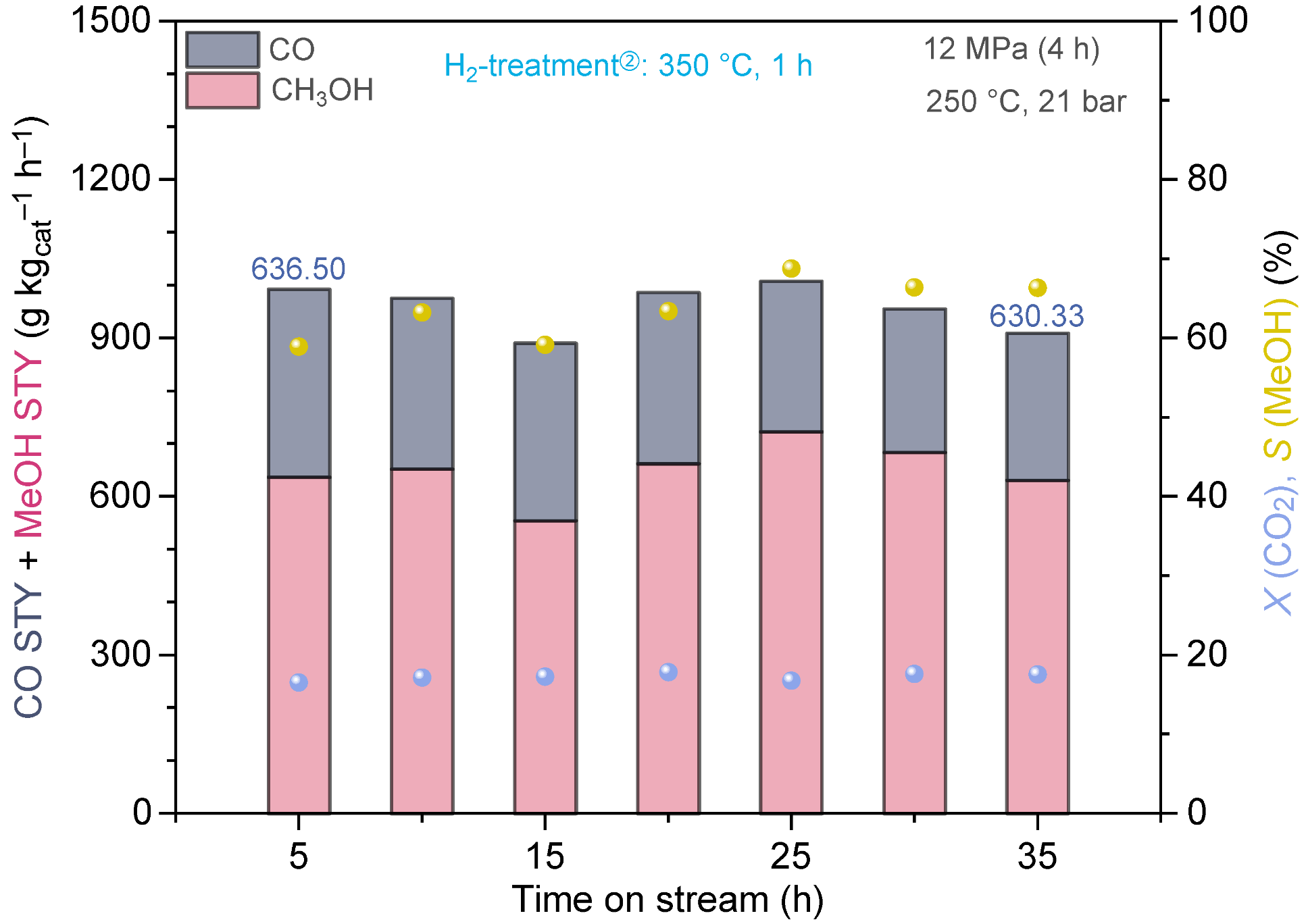


**Figure S3.** Stability test of the 12 MPa (4 h) catalyst. The catalyst was pretreated *in situ* with H_2_ at 350 °C for 1 h. Reaction conditions: molar ratio CO_2_:H_2_ = 1:3, 20 mg catalyst, flow rate 20 mL min^−1^, 250 °C.


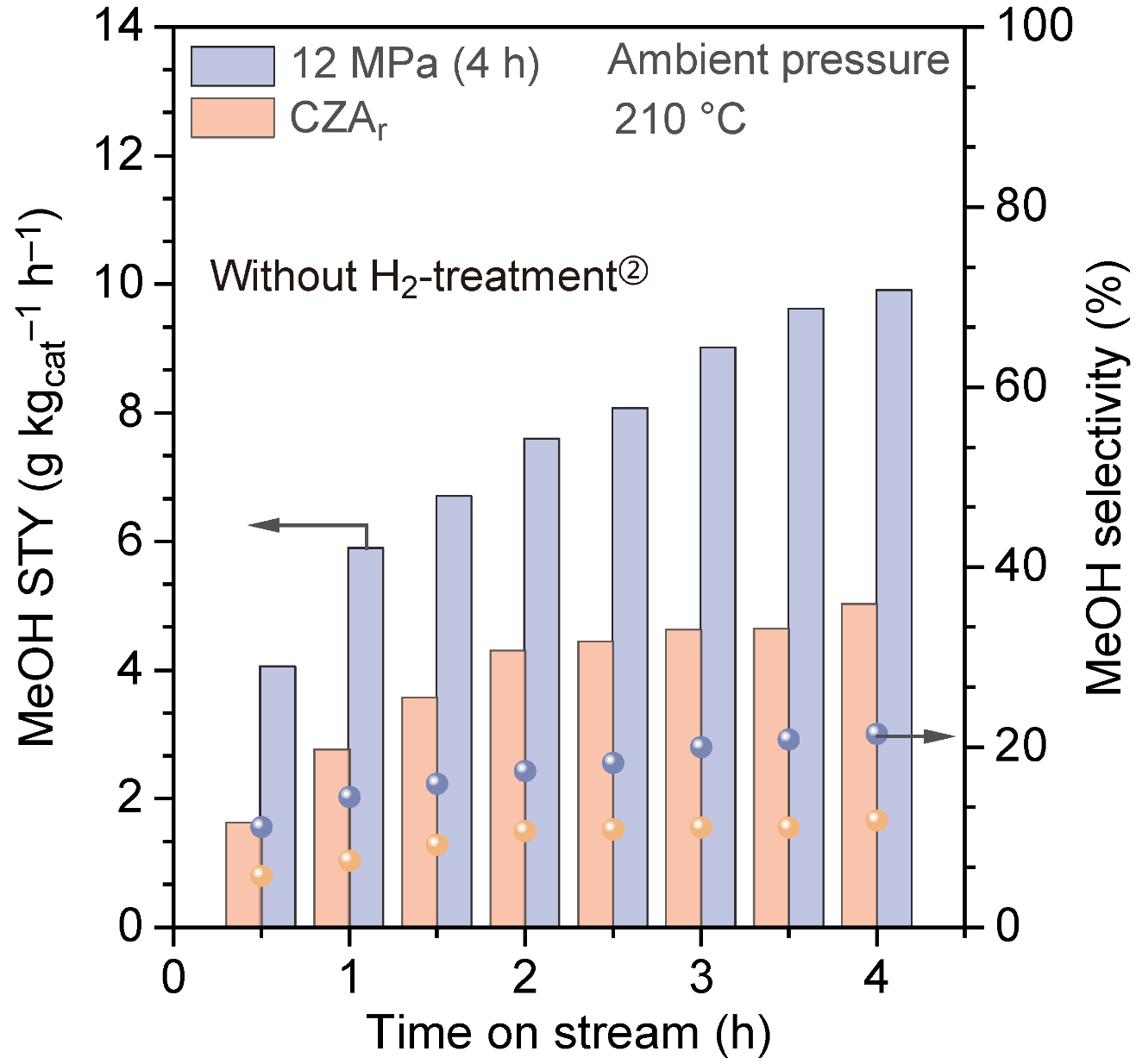


**Figure S4.** Comparative catalytic performances of the CZA_r_ and 12 MPa (4 h) samples without H_2_ treatment**^②^**. The reactions were run at 210 °C and atmospheric pressure.


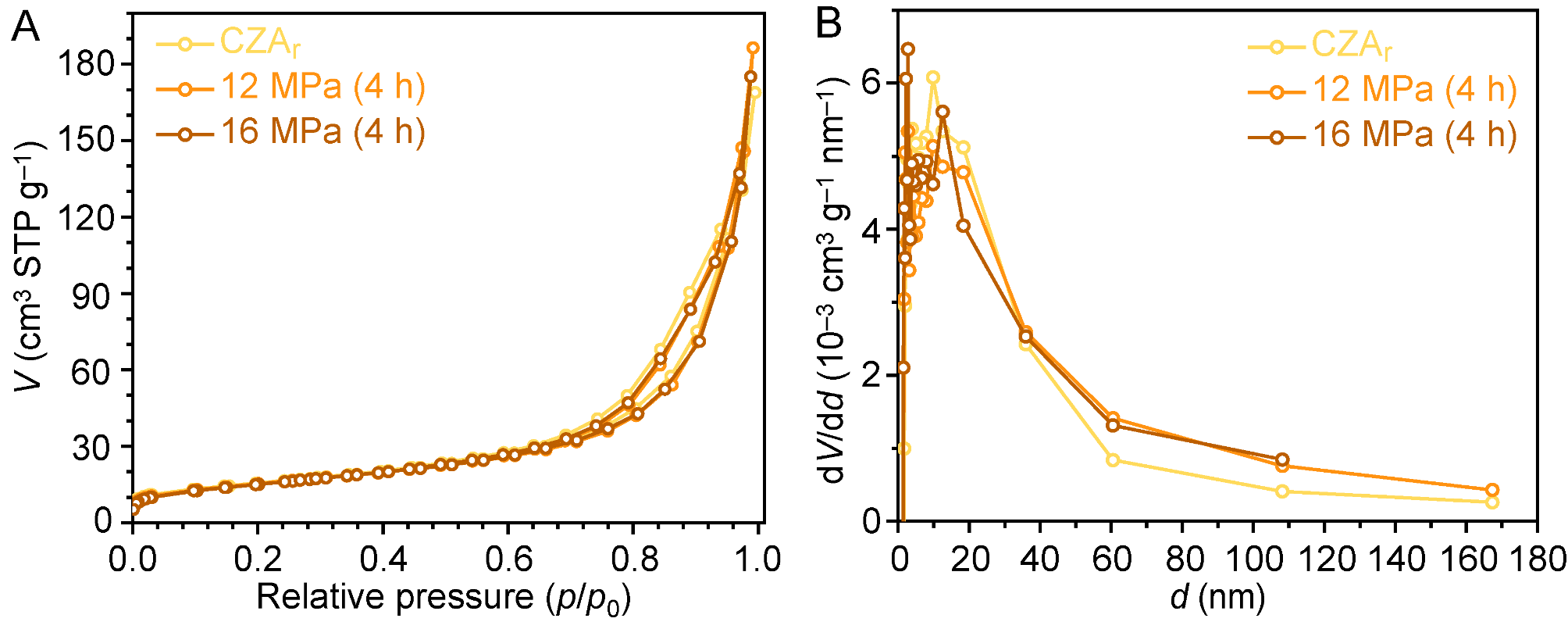


**Figure S5.** Surface area and pore size characterization. A) Nitrogen adsorption-desorption isotherms. B) Pore diameter distributions of the CZA_r_, 12 MPa (4 h), and 16 MPa (4 h) catalysts.


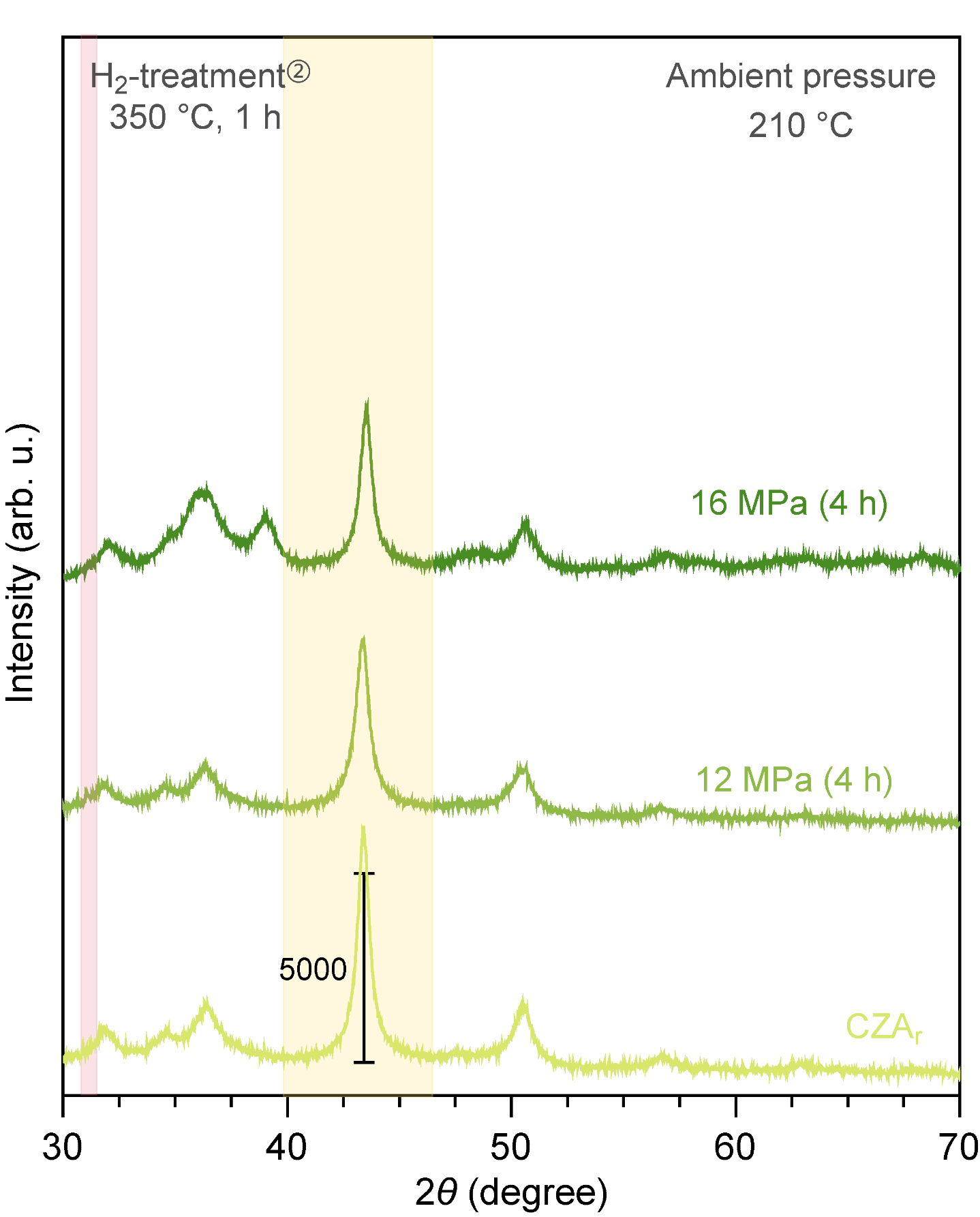


**Figure S6.** XRD patterns for the spent catalysts at 210 °C and atmospheric pressure.


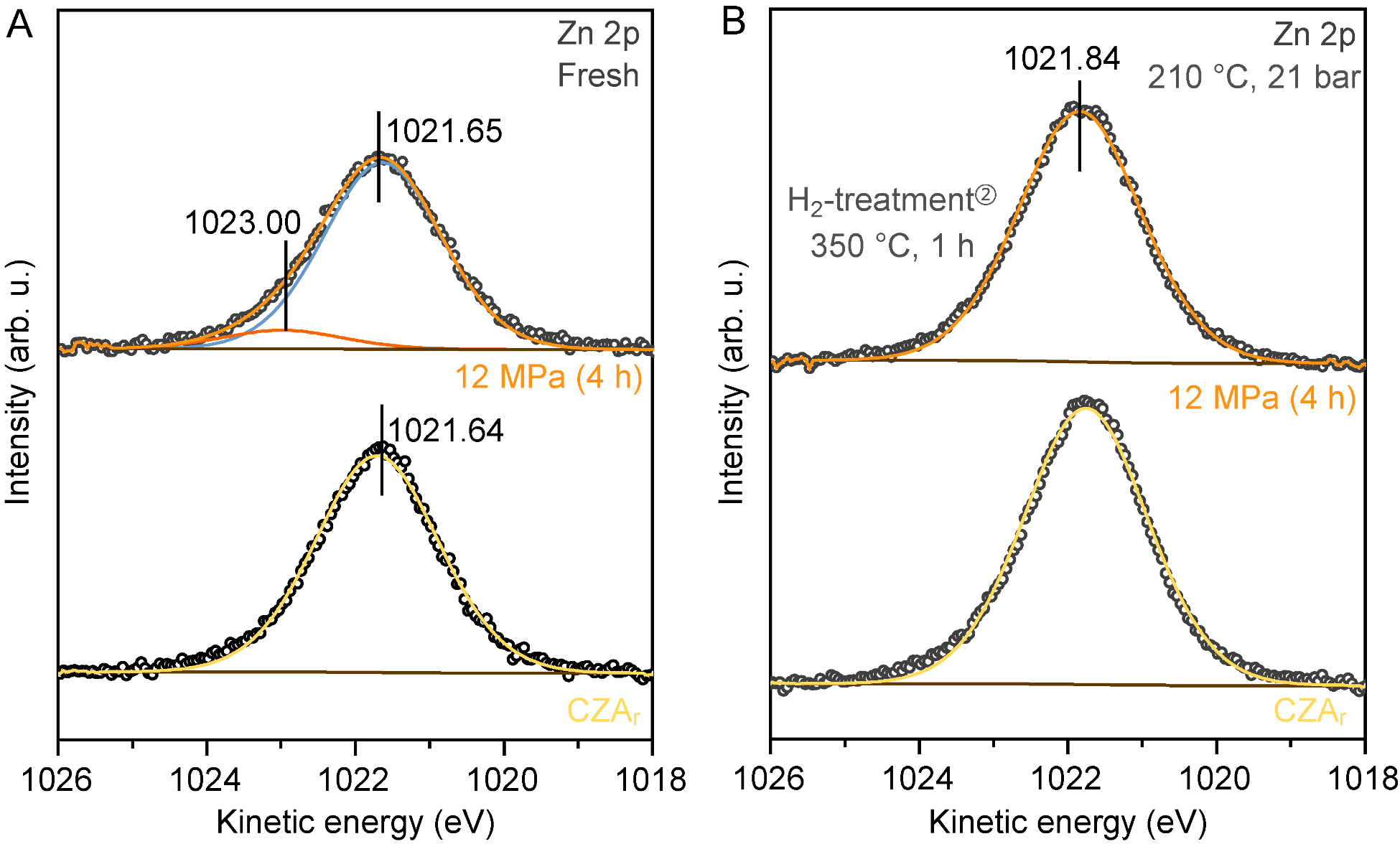


**Figure S7.** Zn 2p_3/2_ XPS results. A) Fresh catalyst. B) Catalyst spent at 210 °C and 21 bar.


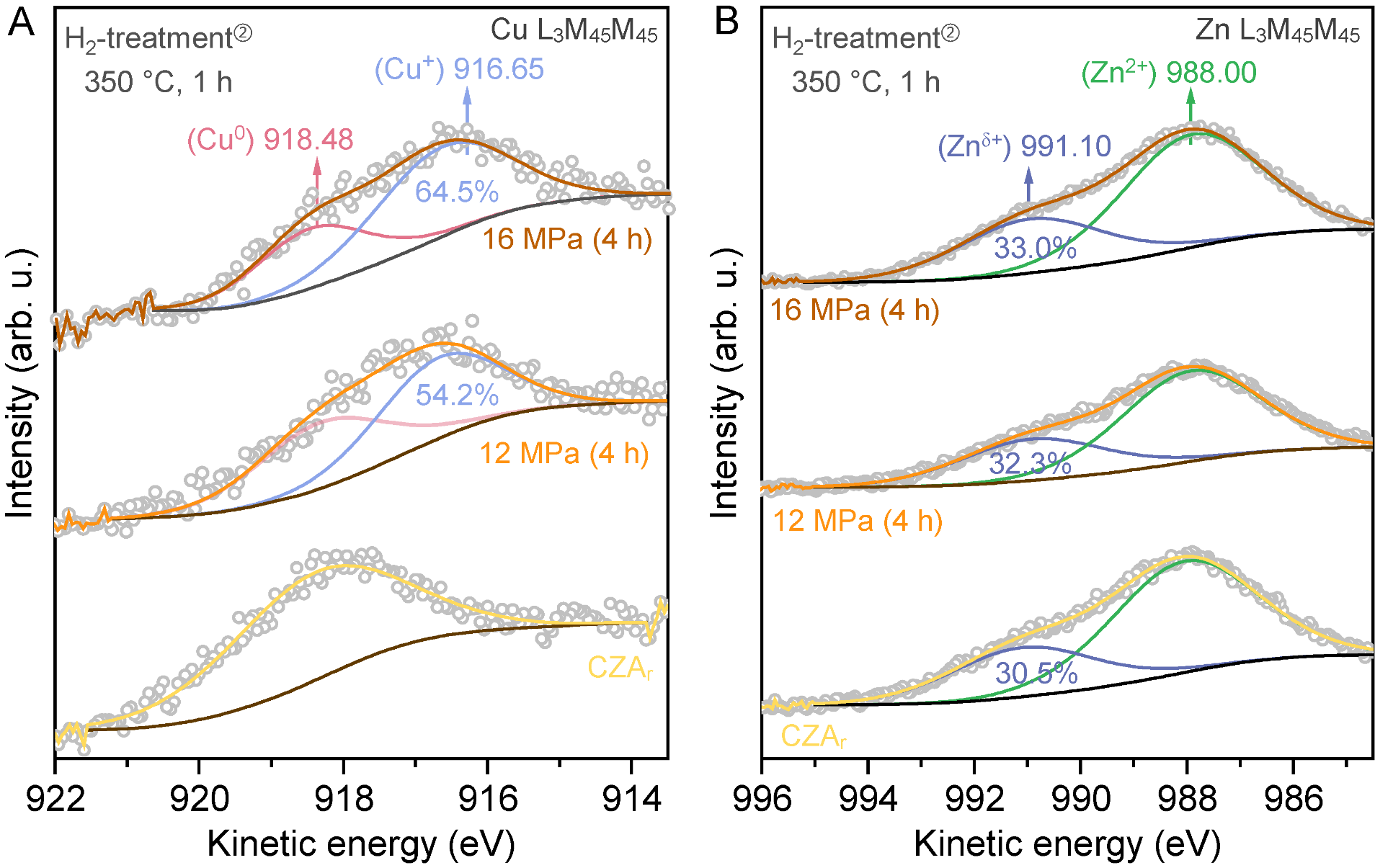


**Figure S8.** Quasi *in situ* XPS Auger spectra of the catalysts after H_2_ treatment**^②^**. A) Cu LMM. B) Zn LMM.


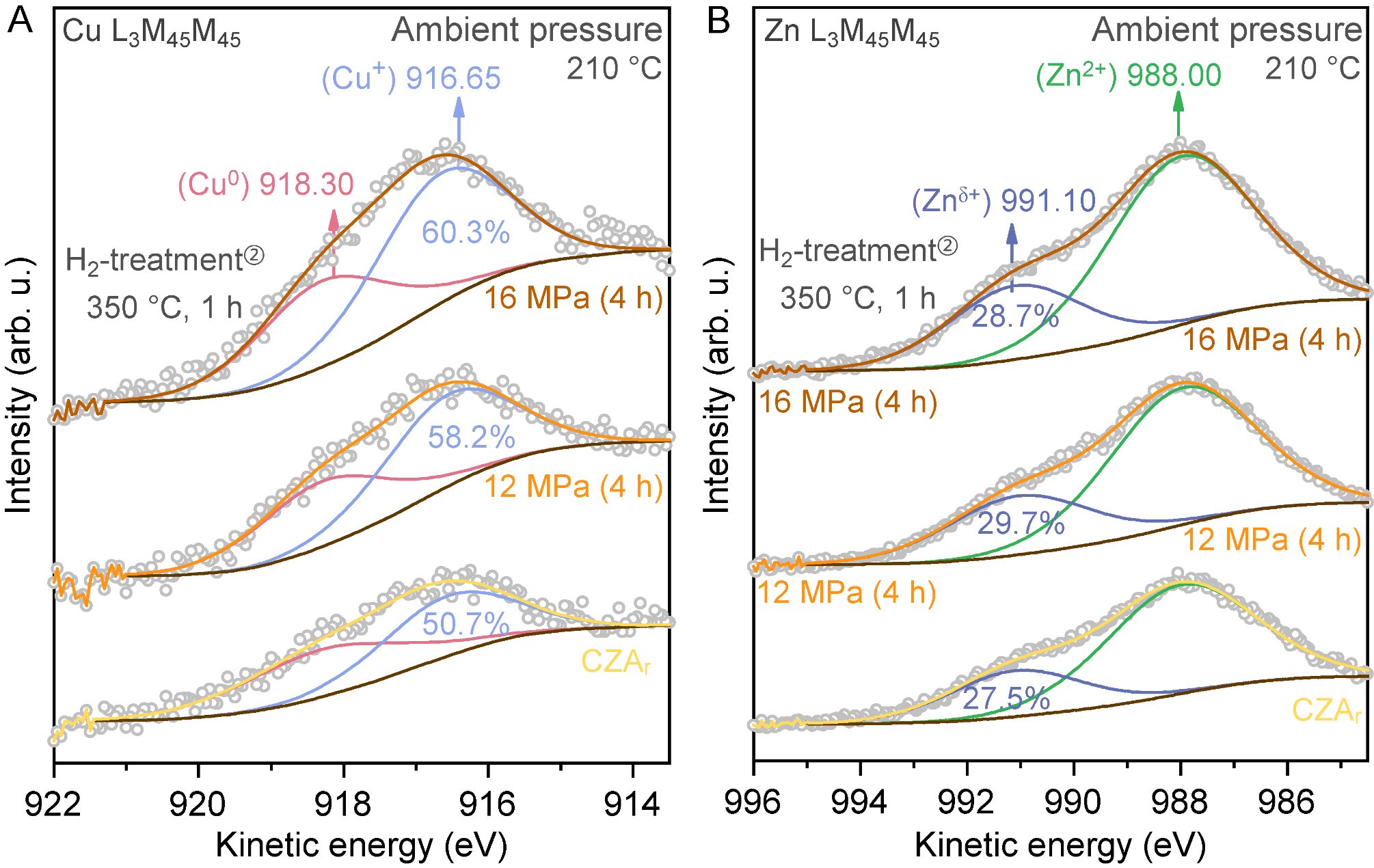


**Figure S9.** Quasi *in situ* XPS Auger spectra of the samples after reaction at 210 °C and atmospheric pressure. A) Cu LMM. B) Zn LMM.


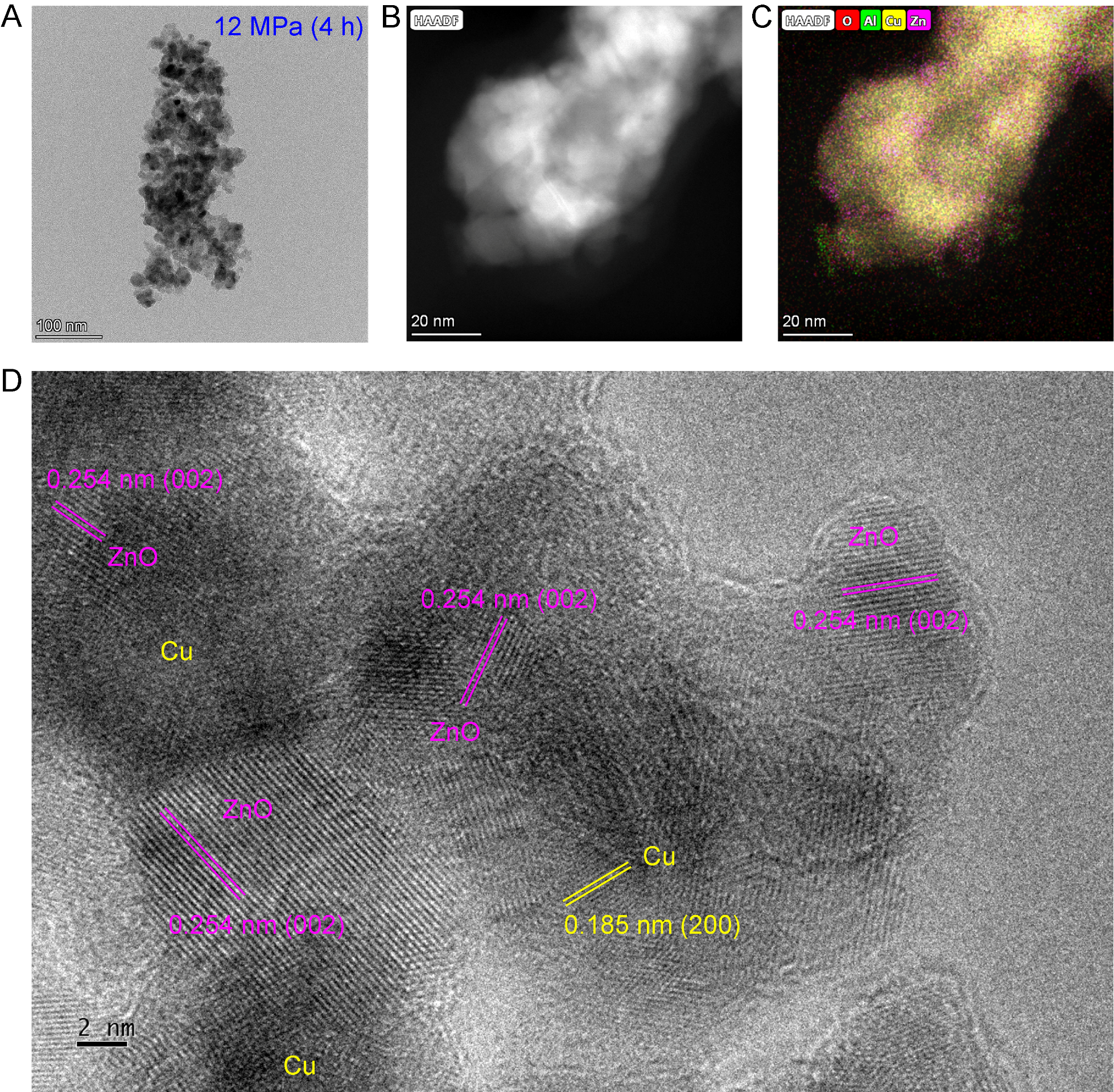


**Figure S10.** TEM characterization of the 12 MPa (4 h) catalyst. A) TEM image. B) HAADF STEM image. C) EDX elemental map. D) High-resolution (HR) TEM image.


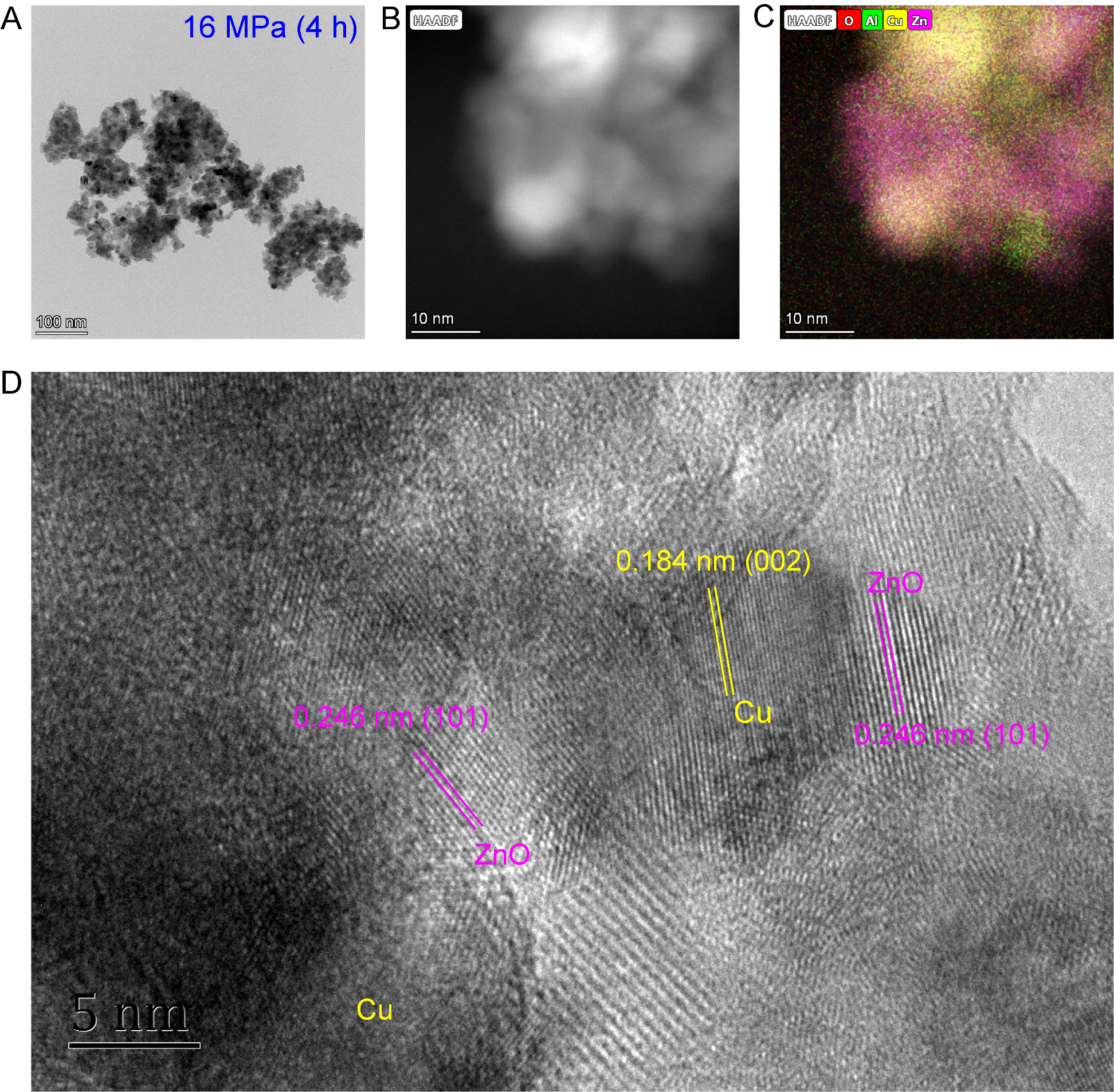


**Figure S11.** TEM characterization of the 16 MPa (4 h) catalyst. A) TEM image. B) HAADF STEM image. C) EDX elemental map. D) HRTEM image.


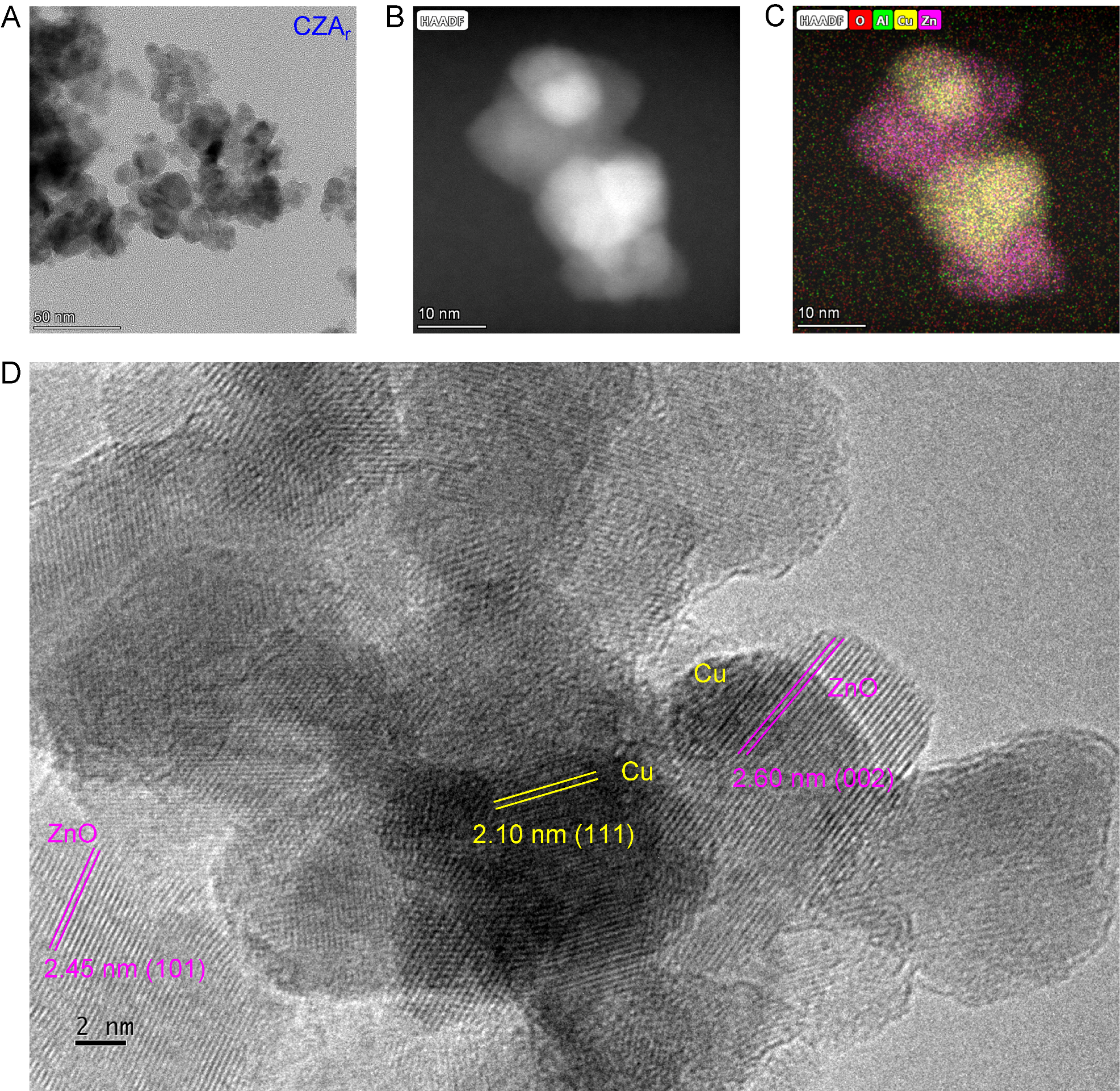


**Figure S12.** TEM characterization of the CZA_r_ catalyst. A) TEM image. B) HAADF STEM image. C) EDX elemental map. D) HRTEM image.


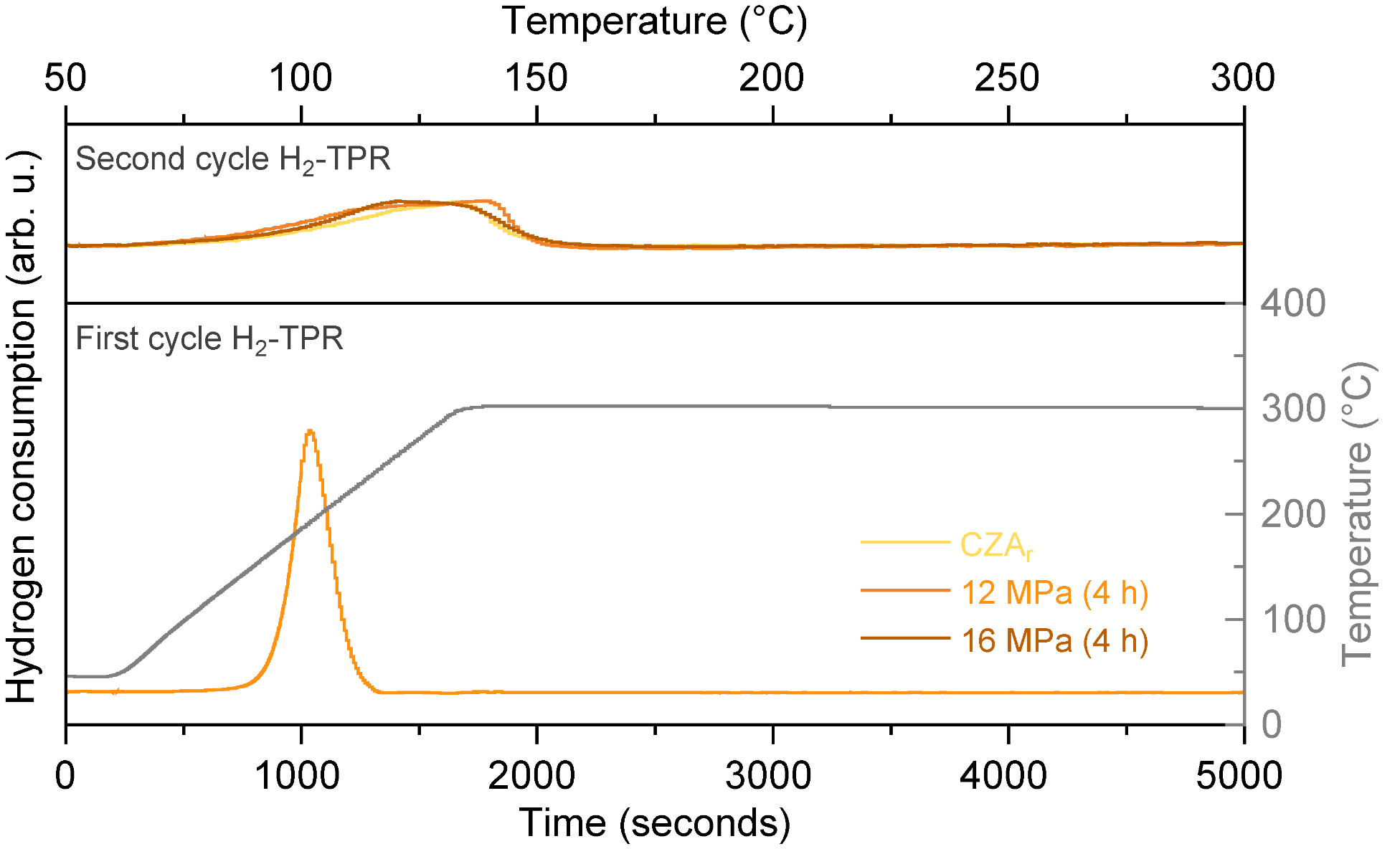


**Figure S13.** First-cycle H_2_-TPR profile of 12 MPa (4 h) (bottom) and H_2_-TPR profiles after the re-oxidation of the activated catalysts with N_2_O (upper).


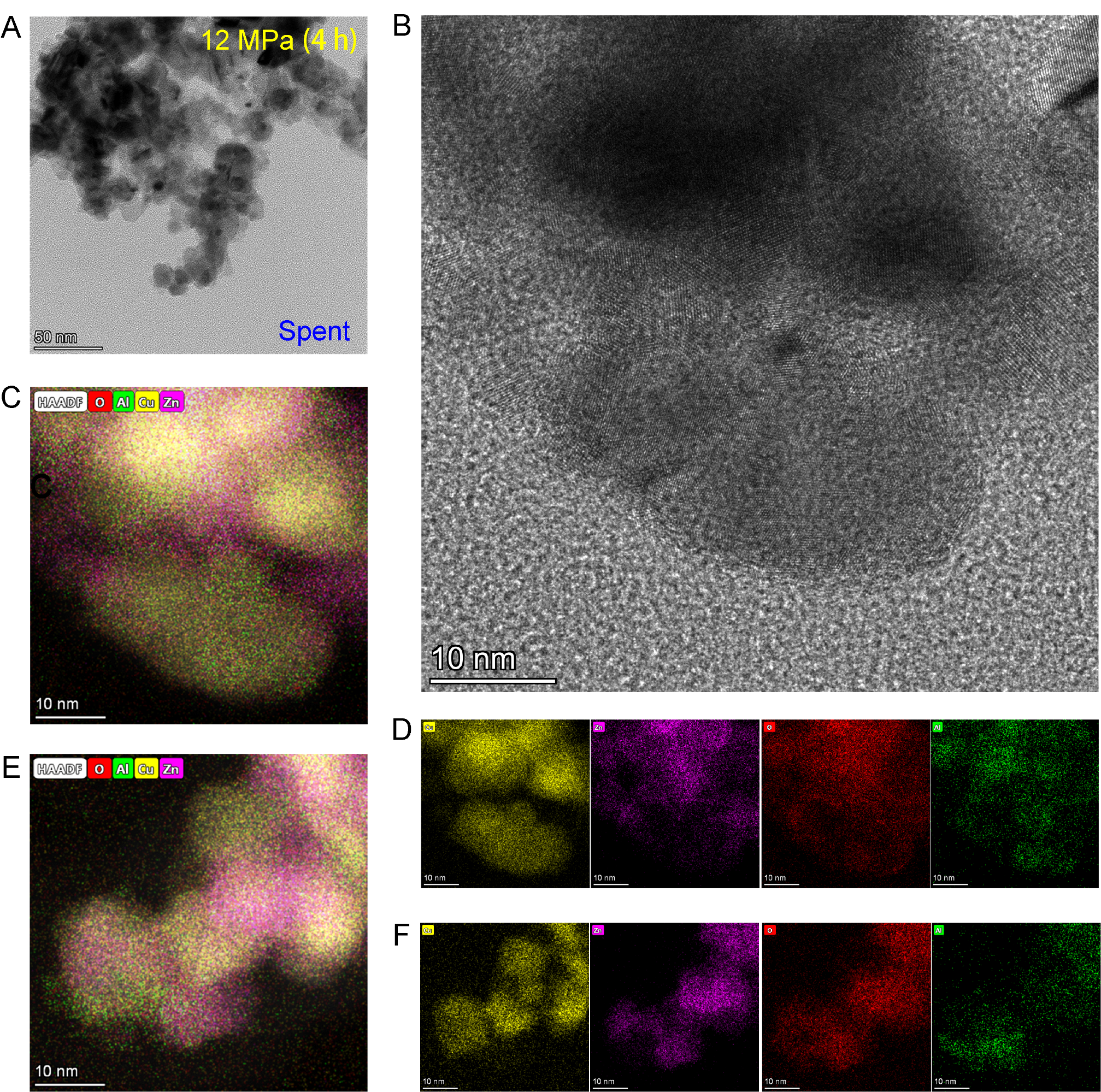


**Figure S14.** TEM characterization of the spent 12 MPa (4 h) catalyst at 210 °C and 21 bar. A) TEM image. B) HRTEM image. C–F) EDX elemental maps.


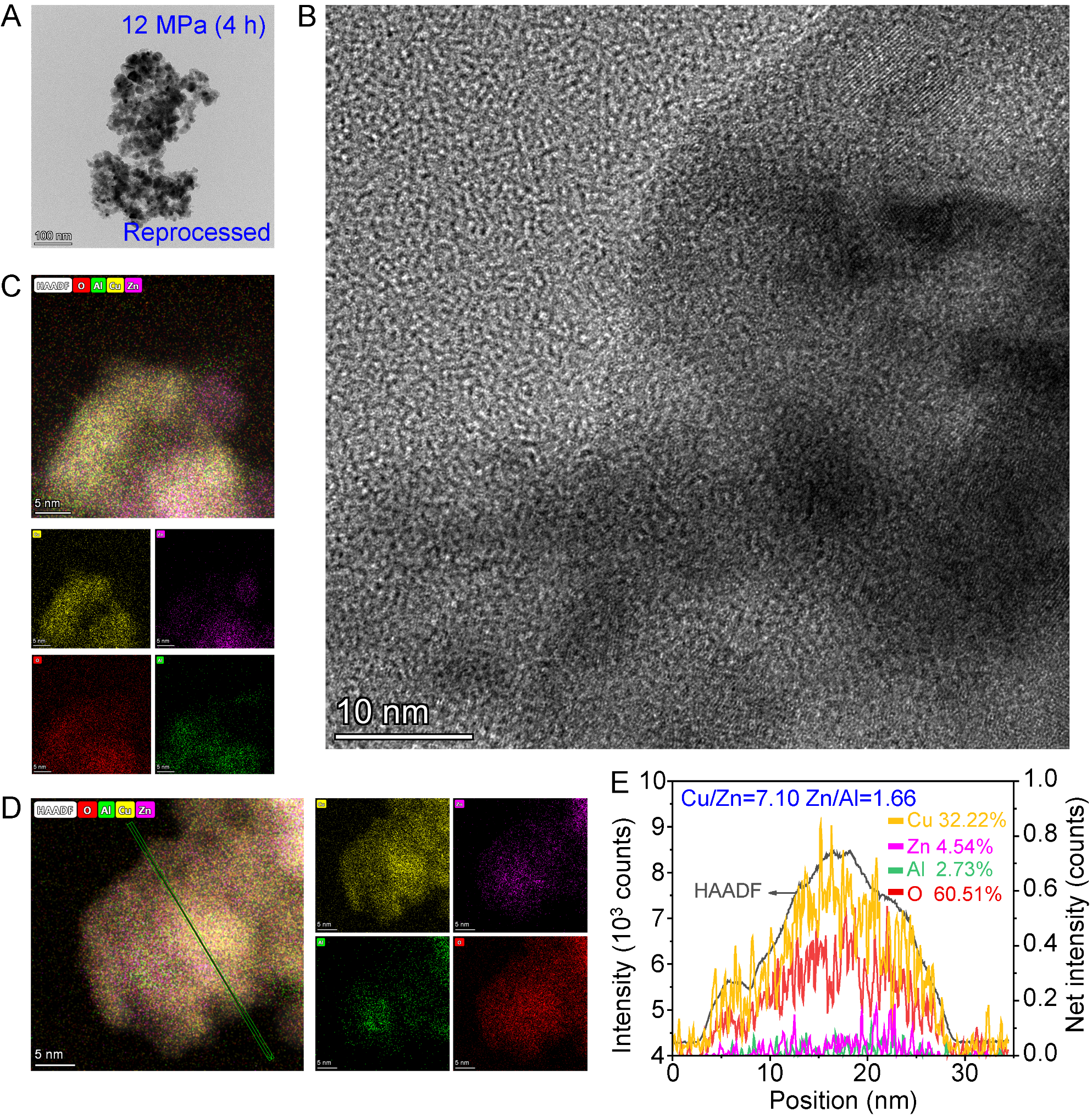


**Figure S15.** TEM characterization of the reprocessed 12 MPa (4 h) catalyst. A) TEM image. B) HRTEM image. C,D) EDX elemental maps. E) EDX line profiles along the green line in (D).


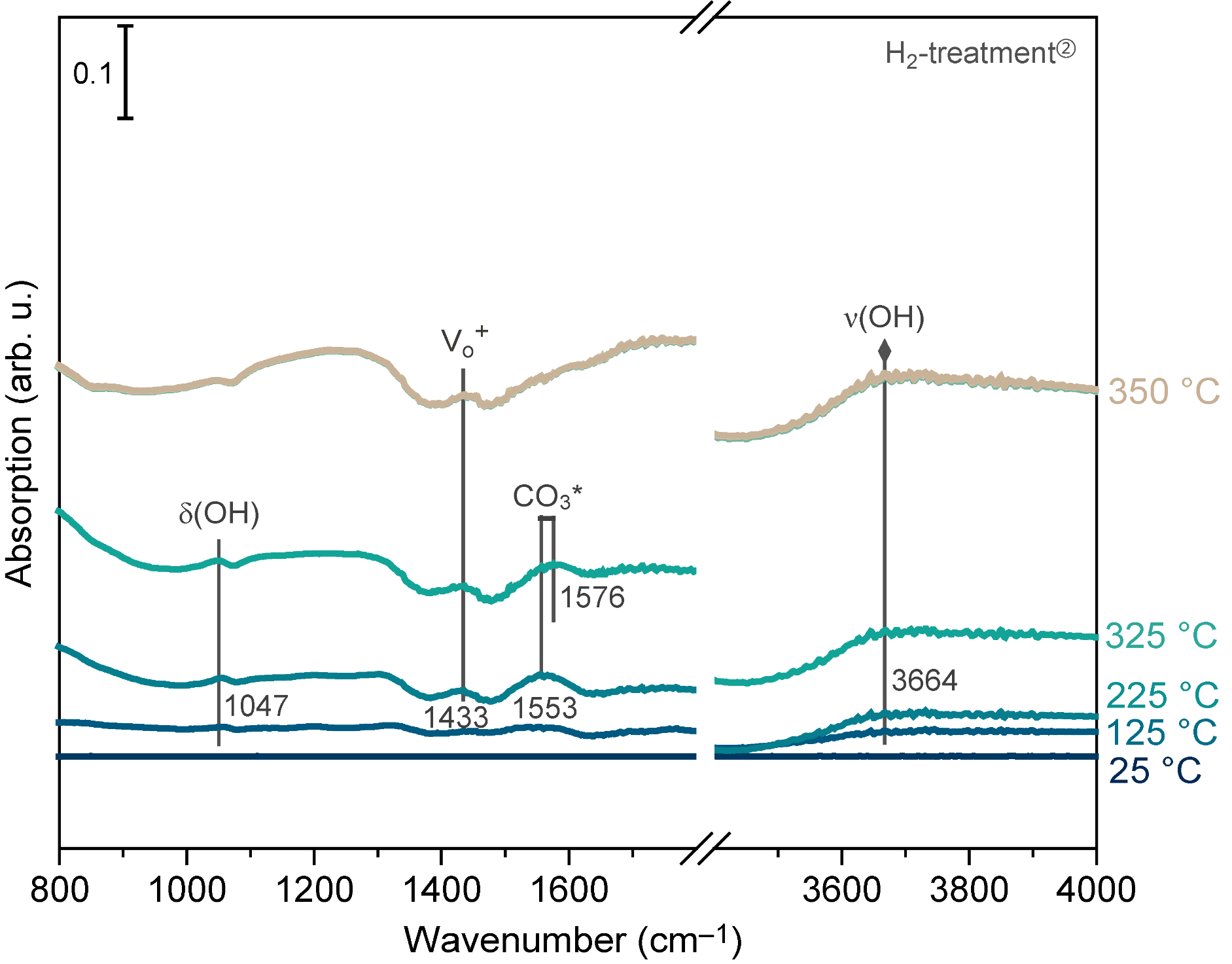


**Figure S16.** *In situ* temperature-programmed DRIFTS of the CZA_r_ sample with the spectra collected when the temperature was increased to 350 °C at a ramping rate of 5 °C min^−1^ in H_2_.


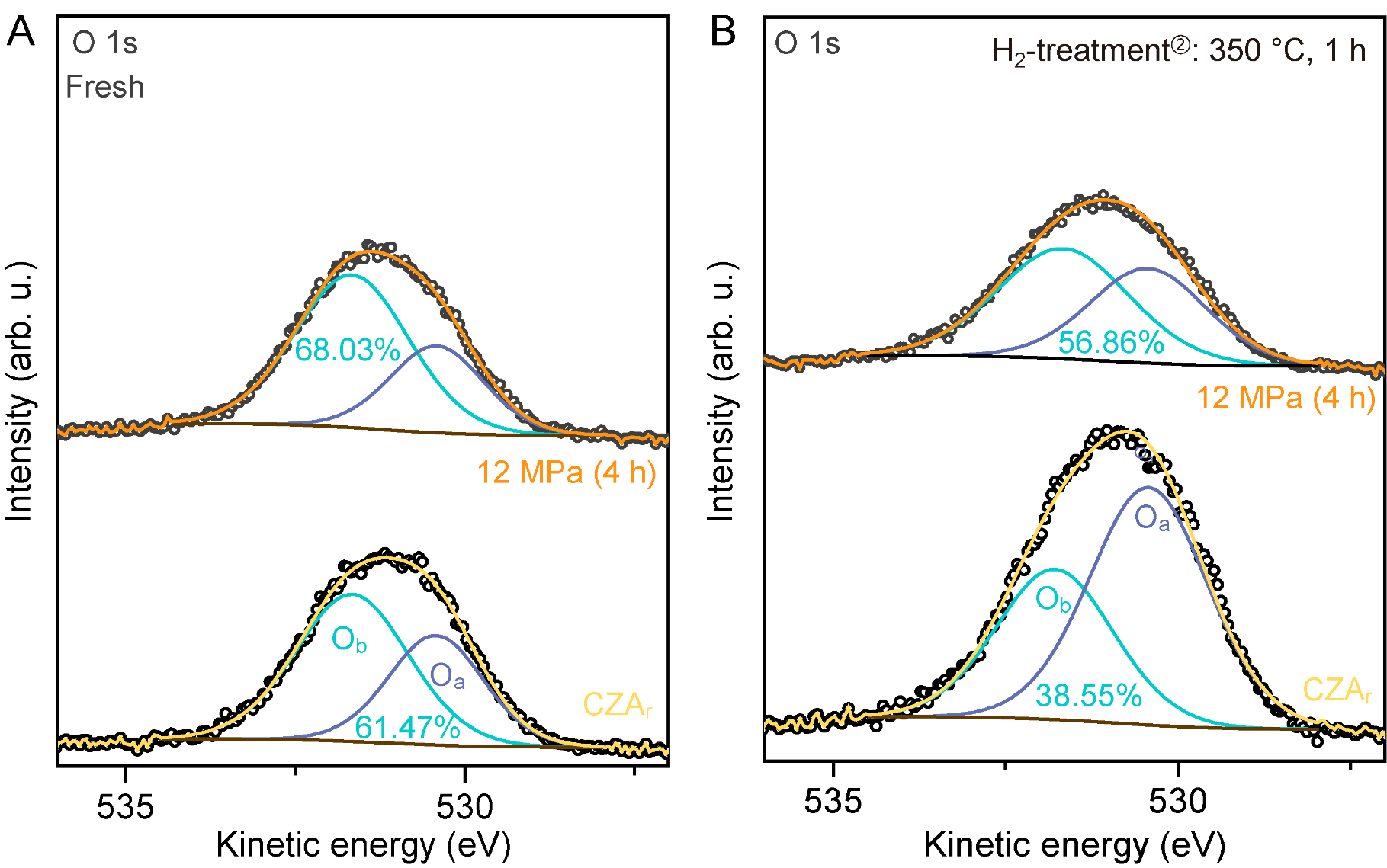


**Figure S17.** O 1s XPS of the catalysts. A) Before H_2_ treatment**^②^**. B) After H_2_ treatment**^②^**. O_a_: lattice oxygen; O_b_: surface chemisorbed oxygen from defect-oxide and hydroxyl-like groups.


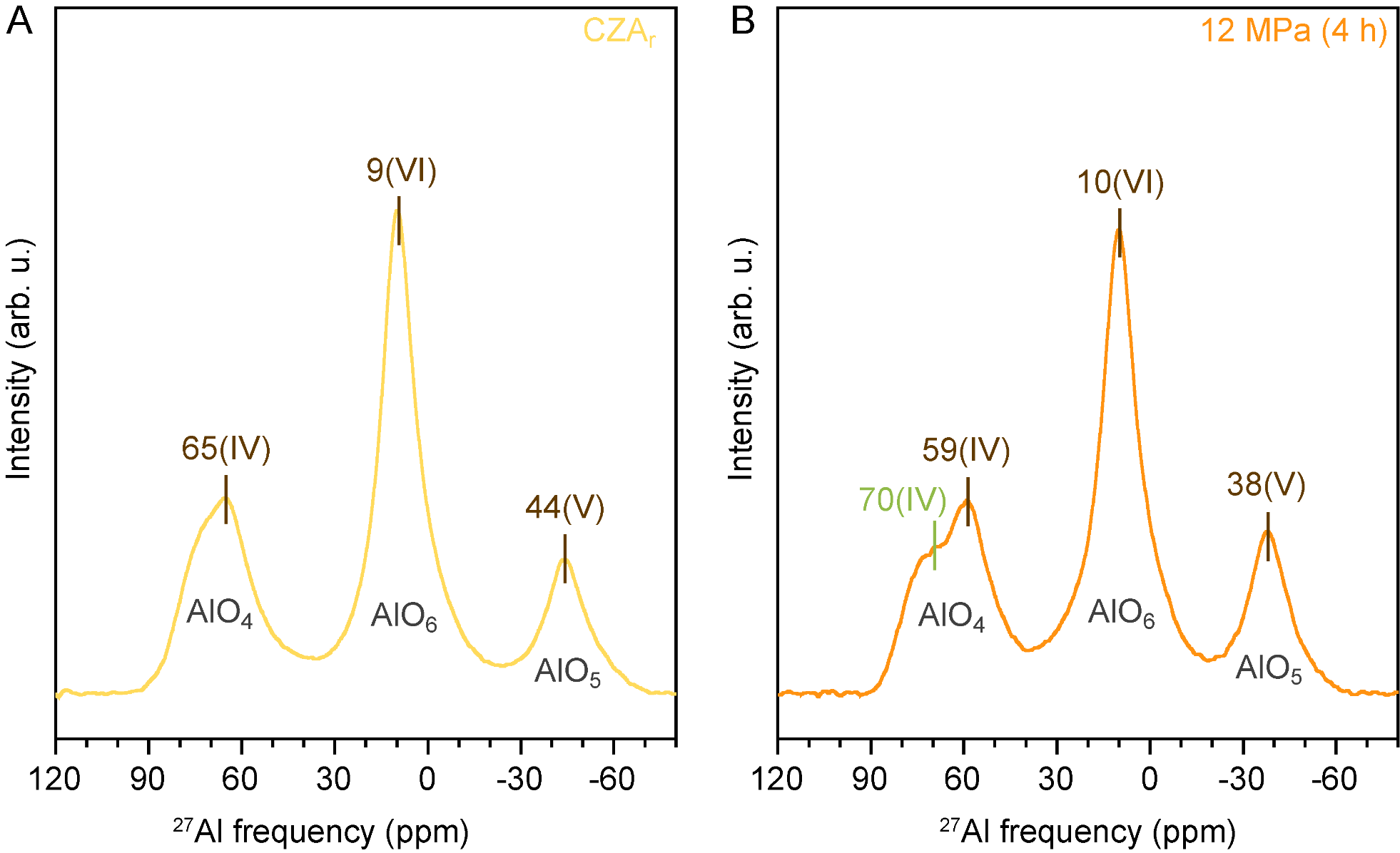


**Figure S18.** ^27^Al magic-angle spinning nuclear magnetic resonance (MAS NMR) spectra. A) CZA_r_. B) 12 MPa (4 h) catalyst. The IV, V, and VI sites represent the tetra-coordinate Al atoms (AlO_4_), penta-coordinate Al atoms (AlO_5_), and octahedral Al atoms (AlO_6_) in the oxide.


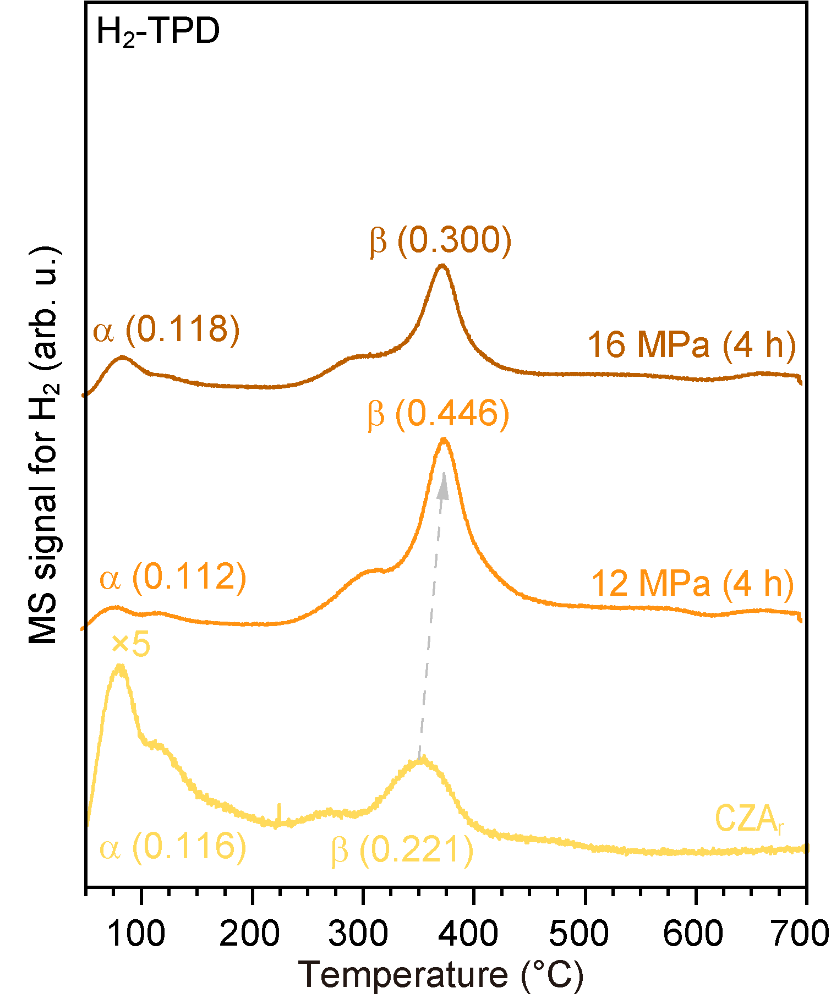


**Figure S19.** H_2_-TPD profiles for all the catalysts.


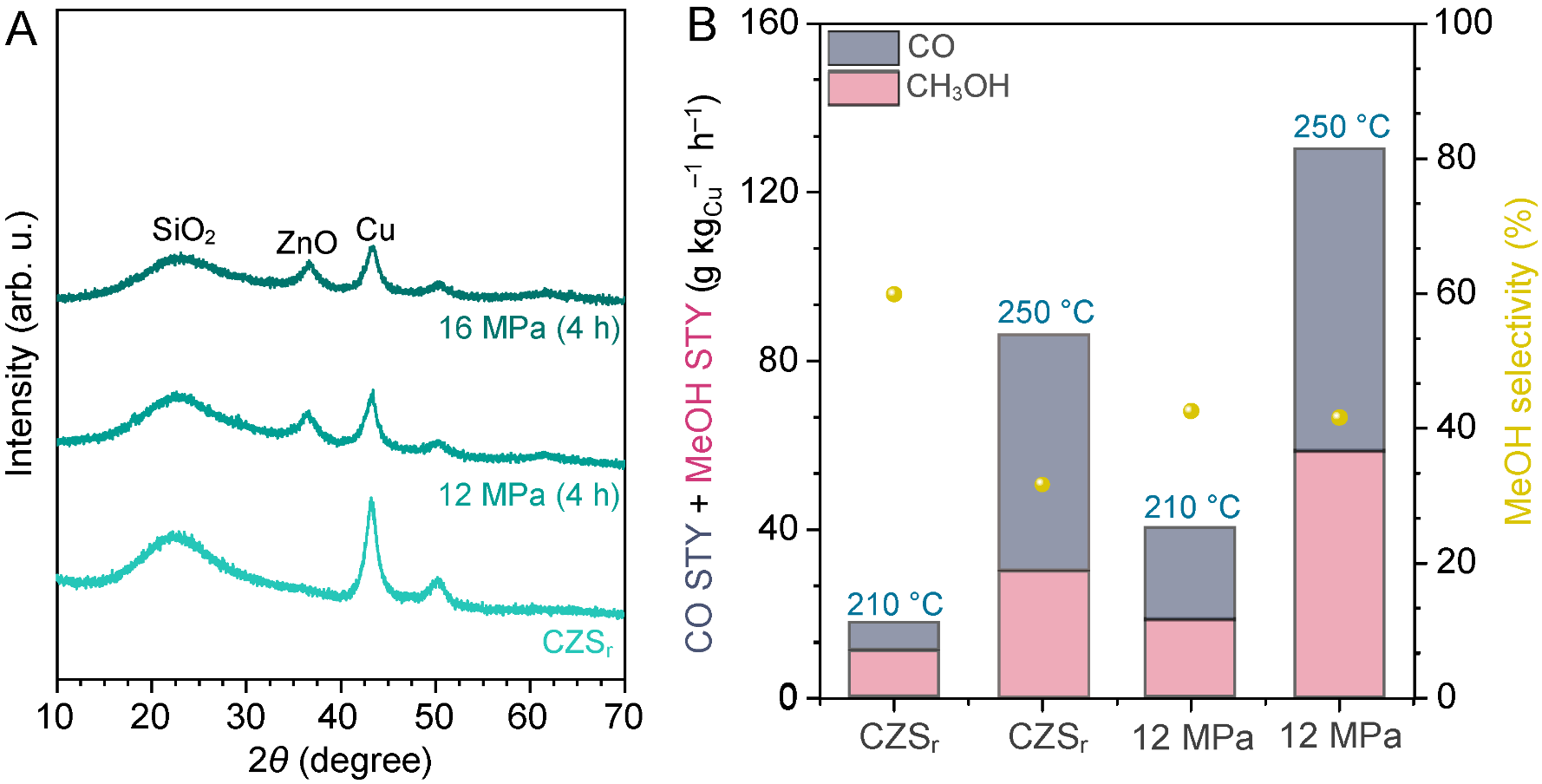


**Figure S20.** Cu/ZnO/SiO_2_ (CZS_r_) catalyst. A) XRD patterns of the fresh CZS_r_ and corresponding activated catalysts treated under different SC CO_2_ pressures. B) Performances of the CZS_r_ and 12 MPa (4 h) catalysts in CO_2_ hydrogenation to methanol. The activities of the catalysts were tested at 21 bar (210 and 250 °C). The MeOH STY of the 12 MPa (4 h) sample (58.47 g kg_Cu_^−1^ h^−1^) increased approximately by two folds, with a higher selectivity (42%), in comparison to its pristine counterpart (29.67 g kg_Cu_^−1^ h^−1^) at the reaction temperature of 250 °C.


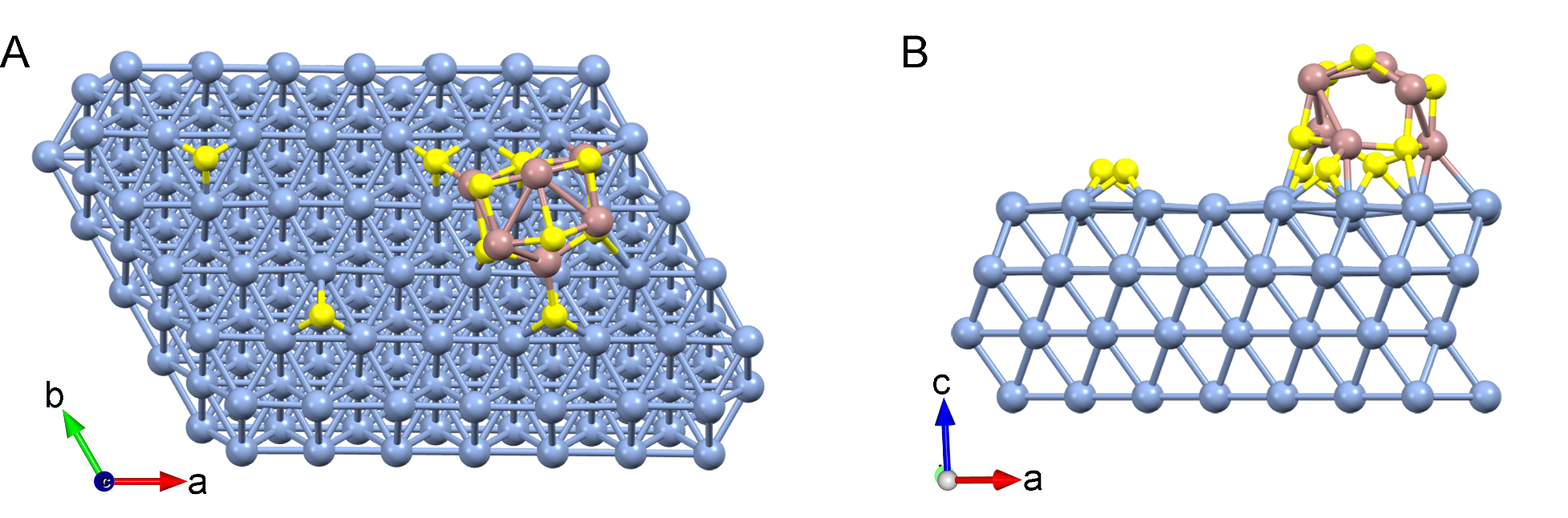


**Figure S21.** DFT calculation model. A) Structure viewed from the *c*-axis, B) Structure viewed from the *b*-axis.


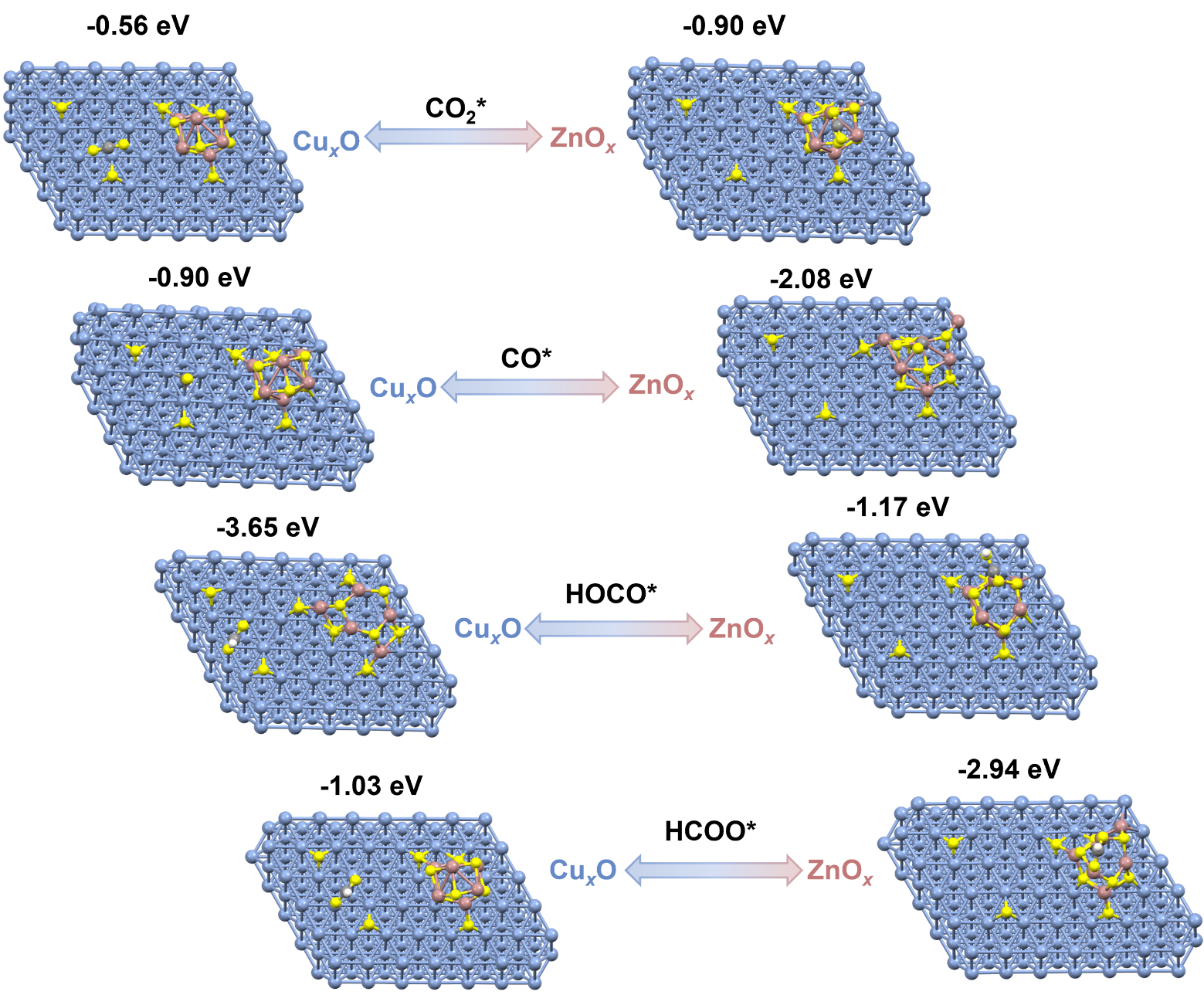


**Figure S22.** DFT-optimized geometries of CO_2_ and key intermediates adsorbed on the different sites of the activated catalyst viewed from the *c*-axis. The left ones belong to Cu*_x_*O and the right ones belong to Cu–ZnO*_x_* on the 12 MPa (4 h) catalyst. Cu: blue; Zn: pink; O: yellow; C: grey; and H: white.

**3. Supporting Tables**

**Table S1.** Performance comparison between the activated CZA_r_ catalyst from this study and other reported Cu-based catalysts for methanol production from CO_2_ hydrogenation.

| Catalyst | Gas hourly space velocity (mL g_cat_^−1^ h^−1^) | *T* (°C) | | *P* (bar) | | MeOH STY  (g kg_cat_^−1^ h^−1^) | | MeOH sel. (%) | | Ref. | |
| --- | --- | --- | --- | --- | --- | --- | --- | --- | --- | --- | --- |
| 12 MPa (4 h) | 13143 | 190 | | 21 | 107 | | 95 | | This work | |  |
|  |  | 210 | | 21 | 259 | | 89 | |  |  |  |
|  |  | 250 | | 21 | 629 | | 64 | |  |  |  |
| CZA_r_ (this work) |  | 210 | 21 | | 126 | | 77 | |  | |  |
| CZA | 15000 | 260 | 50 | | 410 | | 41 | | Ref. 39 | |  |
| CZA | 8758 | 250 | 21 | | 224 | | 56 | | Ref. 18 | |  |
| CZA | 12000 | 250 | 50 | | 340 | | 40 | | Ref. 40 | |  |
| CZA | 18000 | 250 | 50 | | 370 | | 55 | | Ref. 41 | |  |
| CZA | 10000 | 230 | 50 | | 250 | | 58 | | Ref. 42 | |  |
| Cu/Zn/Ga | 18000 | 250 | 45 | | 520 | | 51 | | Ref. 43 | |  |
| La-CZA | N.A. | 250 | 21 | | 69 | | 84 | | Ref. 44 | |  |
| Er_0.2_CuZnO | 6000 | 190 | 50 | | 103 | | 83 | | Ref. 45 | |  |
| Au/CuZnO | 24000 | 200 | 30 | | 164 | | 82 | | Ref. 46 | |  |
| CuZnAl | 8000 | 200 | 30 | | 55 | | 90 | | Ref. 47 | |  |
| CuZrO_2_ | 10000 | 220 | 30 | | 233 | | 77 | | Ref. 48 | |  |
| ZnO_1-_*_x_*/Cu | 14400 | 240 | 40 | | 630 | | 87 | | Ref. 9a | |  |
| CuZnAl | 57600 | 240 | 40 | | 730 | | 83 | | Ref. 9a | |  |
| CZA | 60000 | 260 | 15 | | 540 | | 48 | | Ref. 49 | |  |
| Pd–Cu/Zn | 10800 | 230 | 45 | | 120 | | 78 | | Ref. 50 | |  |

**Table S2.** Brunauer-Emmett-Teller (BET) surface areas (*S*_BET_), total pore volumes, average pore sizes, Cu/Zn contents, Cu dispersions (*D*_Cu_), and Cu surface areas of the activated Cu/ZnO/Al_2_O_3_ catalysts.

| Sample | *S*_BET_ (m^2^ g^−1^)^a^ | Volume (cm^3^ g^−1^)^b^ | Average pore diameter (nm)^c^ | Cu content (wt%)^d^ | Zn content (wt%)^d^ | Cu diameter (nm)^e^ | Cu dispersion (%)^e^ | Cu surface area (m^2^ g^−1^)^e^ |
| --- | --- | --- | --- | --- | --- | --- | --- | --- |
| CZA_r_ | 54.85 | 0.25 | 22.96 | 62.35 | 29.83 | 9.26 | 10.80 | 73.08 |
| 12 MPa (4 h) | 55.11 | 0.28 | 23.43 | 58.89 | 30.02 | 7.72 | 12.95 | 87.61 |
| 16 MPa (4 h) | 54.11 | 0.27 | 19.28 | 57.92 | 28.78 | 9.62 | 10.40 | 70.37 |

^a)^The BET surface areas were calculated according to the BET method.

^b)^Total pore volume.

^c)^Average pore size based on the BJH method.

^d)^The Cu and Zn contents were determined by ICP-OES.

^e)^The diameters, dispersions, and surface areas of Cu were calculated from N_2_O reactive frontal chromatography.

**Table S3.** Calculated adsorption energies in eV on the different sites using VASP.

| Activation site | Species | *E* (total) | *E* (slab) | *E* (reference) | Adsorption energy |
| --- | --- | --- | --- | --- | --- |
| Cu*_x_*O | CO_2_* | -469.07 | -446.18 | -22.33 | -0.56 |
|  | CO* | -461.96 | -446.18 | -14.87 | -0.90 |
|  | HOCO* | -475.55 | -446.18 | -25.71 | -3.65 |
|  | HCOO* | -472.93 | -446.18 | -25.71 | -1.03 |
| Cu–ZnO*_x_* | CO_2_* | -469.41 | -446.18 | -22.33 | -0.90 |
|  | CO* | -463.13 | -446.18 | -14.87 | -2.08 |
|  | HOCO* | -473.06 | -446.18 | -25.71 | -1.17 |
|  | HCOO* | -474.83 | -446.18 | -25.71 | -2.94 |

**4. Supporting References**

[39] J. Hu, L. Yu, J. Deng, Y. Wang, K. Cheng, C. Ma, Q. Zhang, W. Wen, S. Yu, Y. Pan, *Nat. Catal.* **2021**, *4*, 242.

[40] P. Gao, F. Li, N. Zhao, F. Xiao, W. Wei, L. Zhong, Y. Sun, *Appl. Catal. A: Gen.* **2013**, *468*, 442–452.

[41] B. An, J. Zhang, K. Cheng, P. Ji, C. Wang, W. Lin, *J. Am. Chem. Soc.* **2017**, *139*, 3834.

[42] P. Gao, L. Zhong, L. Zhang, H. Wang, N. Zhao, W. Wei, Y. Sun, *Catal. Sci. Technol.* **2015**, *5*, 4365.

[43] M. M.-J. Li, C. Chen, T. Ayvalı, H. Suo, J. Zheng, I. F. Teixeira, L. Ye, H. Zou, D. O’Hare, S. C. E. Tsang, *ACS Catal.* **2018**, *8*, 4390.

[44] B. Xie, P. Kumar, T. H. Tan, A. A. Esmailpour, K.-F. Aguey-Zinsou, J. Scott, R. Amal, *ACS Catal.* **2021**, *11*, 5818.

[45] C. Huang, S. Zhang, W. Wang, H. Zhou, Z. Shao, L. Xia, H. Wang, Y. Sun, *ACS Catal.* **2024**, *14*, 1324.

[46] G. Xie, R. Jin, P. Ren, Y. Fang, R. Zhang, Z. Wang, *Appl. Catal. B: Environ.* **2023**, *324*, 122233.

[47] F. Zhao, L. Fan, K. Xu, D. Hua, G. Zhan, S.-F. Zhou, *J. CO_2_ Util.* **2019**, *33*, 222.

[48] W. Wang, Z. Qu, L. Song, Q. Fu, *J. Energy Chem.* **2020**, *40*, 22.

[49] M. Zabilskiy, V. L. Sushkevich, M. A. Newton, J. A. van Bokhoven, *ACS Catal.* **2020**, *10*, 14240.

[50] B. Hu, Y. Yin, G. Liu, S. Chen, X. Hong, S. C. E. Tsang, *J. Catal.* **2018**, *359*, 17.
